# Correction: Sure-thing vs. probabilistic charitable giving: Experimental evidence on the role of individual differences in risky and ambiguous charitable decision-making

**DOI:** 10.1371/journal.pone.0307847

**Published:** 2024-07-22

**Authors:** Miguel Costa-Gomes, Philipp Schoenegger

The authors are listed out of order. Please view the correct author order, affiliations, and citations here:

Miguel Costa-Gomes^1^, Philipp Schoenegger^2^

**1** School of Economics and Finance & School of Philosophical, Anthropological and Film Studies, University of St Andrews, St Andrews, Scotland, United Kingdom**, 2** School of Economics and Finance, University of St Andrews, St Andrews, Scotland, United Kingdom

Costa-Gomes M, Schoenegger P (2022) Sure-thing vs. probabilistic charitable giving: Experimental evidence on the role of individual differences in risky and ambiguous charitable decision-making. PLoS ONE 17(9): e0273971. https://doi.org/10.1371/journal.pone.0273971
