# Supplementary material for: Sure-thing vs. probabilistic charitable giving: Experimental evidence on the role of individual differences in risky and ambiguous charitable decision-making
Source: PLoS One. 2022 Sep 22;17(9):e0273971. doi: 10.1371/journal.pone.0273971 (PMC9499298; doi:10.1371/journal.pone.0273971)
Supplement: S1 Appendix — (PDF) [file pone.0273971.s001.pdf]

Appendix A – Preregistered Model Specification for Model (1)

In Appendix Table 1, Model (4), we present Model (1) as we initially pre-registered it without controlling for amount donated. We do not find a difference in results.

APPENDIX TABLE 1—REGRESSION RESULTS FOR CHARITABLE GIVING BEHAVIOUR IN MAIN CHOICE
PREDICTING CHOICE BETWEEN SURE-THING AND PROBABILISTIC CHARITIES

	(4)
Risk Attitude	.001 (.005)
Ambiguity Aversion	-.002 (.005)
Numeracy	.003 (.024)
Empathy	-.001 (.002)
Optimism	-.005 (.003)
Donor Type	
Warm-Glow	-.020 (.051)
Pure Altruism	-.056 (.064)
Age	.003 (.002)
Gender	.014 (.050)
Education	
Undergraduate degree	.039 (.051)
Postgraduate/Professional degree	-.019 (.056)
Religion	
Protestantism	-.077 (.067)
Catholicism	.011 (.074)
Islam	-.114 (.122)
Judaism	-.131 (.379)
Buddhism	-.133 (.287)
Hinduism	.345 (.276)
Religious Participation	.041 (.089)
Marriage Status	.043 (.050)
Children	-.004 (.054)
Financial Wellbeing	.005 (.024)
Employment	
Out of the workforce	-.159 (.097)
Part-time employment	.001 (.085)
Full-time employment	-.060 (.079)
R^2	.055
Sample size	307

Notes: OLS regression reporting unstandardised coefficients and standard errors. Outcome variable is charity choice (0 = sure-thing charity, 1 = probabilistic charity). *p<.1, **p<.05, ***p<.01, ****p<.001
