# Supplementary material for: Sure-thing vs. probabilistic charitable giving: Experimental evidence on the role of individual differences in risky and ambiguous charitable decision-making
Source: PLoS One. 2022 Sep 22;17(9):e0273971. doi: 10.1371/journal.pone.0273971 (PMC9499298; doi:10.1371/journal.pone.0273971)
Supplement: S2 Appendix — (PDF) [file pone.0273971.s002.pdf]

Appendix B – Robustness Checks for Main Choice

In Appendix Table 2, Model (5), we present a robustness check for Model (1) that runs the OLS model as a logit model. We find the same pattern of results throughout.

APPENDIX TABLE 2—REGRESSION RESULTS FOR MAIN CHOICE - LOGIT ROBUSTNESS CHECK
PREDICTING CHOICE BETWEEN SURE-THING AND PROBABILISTIC CHARITIES

	(5)
Risk Attitude	.002 (.044)
Ambiguity Aversion	-.033 (.037)
Numeracy	.054 (.192)
Empathy	.001 (.020)
Optimism	-.035 (.026)
Donor Type	
Warm-Glow	-.157 (.401)
Pure Altruism	-.499 (.554)
Age	.024 (.016)
Gender	.084 (.401)
Education	
Undergraduate degree	.292 (.398)
Postgraduate/Professional degree	-.176 (.453)
Religion	
Protestantism	-.673 (.579)
Catholicism	.003 (.541)
Islam	-1.074 (1.193)
Judaism	-19.179 (40192.970)
Buddhism	-19.198 (28374.585)
Hinduism	1.886 (1.651)
Religious Participation	.407 (.722)
Marriage Status	.330 (.388)
Children	-.011 (.427)
Financial Wellbeing	.067 (.195)
Employment	
Out of the workforce	-1.375* (.796)
Part-time employment	-.010 (.634)
Full-time employment	-.463 (.611)
Cox & Snell R^2	.055
Sample size	307

Notes: Log-odds and standard errors. *p<.1, **p<.05, ***p<.01, ****p<.001

We also report an additional robustness check of Model (1) in Appendix Table 3, Model (6). Specifically, we report a random effects model with the stimulus material being treated as a random effect. As we had three sure-thing and three probabilistic charities, there were nine

charity pairs that participants could have been presented with. In Model (6), we treated the stimulus (i.e., the charity pair presented) as a random effect (with nine levels). Because significance levels are not unproblematic in mixed models like this, we also report 95% confidence intervals of the estimates. The results indicate that our null effect is robust to this model choice as well.

APPENDIX TABLE 3— REGRESSION RESULTS FOR MAIN CHOICE – RANDOM EFFECTS ROBUSTNESS CHECK
PREDICTING CHOICE BETWEEN SURE-THING AND PROBABILISTIC CHARITIES

	(6)	
Risk Attitude	.002 (.005)	[-.008, .012]
Ambiguity Aversion	-.002 (.005)	[-.010, .006)
Numeracy	.003 (.023)	[-.042, 0.47]
Empathy	.001 (.002)	[-.005, .004]
Optimism	-.006* (.003)	[-.012, .001]
Donor Type		
Warm-Glow	-.007 (.047)	[-.099, .086]
Pure Altruism	-.040 (.059)	[-.156, .077]
Age	.001 (.002)	[-.003, .004]
Gender	-.024 (.047)	[-.115, .068]
Education		
Undergraduate degree	.041 (.048)	[-.053, .134]
Postgraduate/Professional degree	-.023 (.051)	[-.123, .078]
Religion		
Protestantism	-.043 (.062)	[-.165, .080]
Catholicism	.020 (.068)	[-.114, .154]
Islam	-.035 (.113)	[-.246, .188]
Judaism	-.006 (.354)	[-.691, .703]
Buddhism	-.060 (.265)	[-.581, .462]
Hinduism	.325 (.254)	[-.175, .825]
Religious Participation	-.004 (.083)	[-.168, .159]
Marriage Status	-.019 (.046)	[-.071, .108]
Children	.009 (.050)	[-.090, .107]
Financial Wellbeing	.009 (.022)	[-.035, .053]
Employment		
Part-time employment	.058 (.054)	[-.047, .164]
Full-time employment	.015 (.049)	[-.083, .112]
Sample size	307	

Notes: Random effects model, coefficients, and standard errors, as well as 95% CIs. *p<.1, **p<.05, ***p<.01, ****p<.001

Appendix Table 4, Model (7) presents the main results with the original exclusion criteria, i.e., we exclude variables with less than or equal to one instance of '1'. We find no differences in

results. To maintain an acceptable level of power, all main results in the paper are reported with less strict exclusion criteria.

APPENDIX TABLE 4— REGRESSION RESULTS FOR MAIN CHOICE – STRICTER EXCLUSIONS ROBUSTNESS CHECK
PREDICTING CHOICE BETWEEN SURE-THING AND PROBABILISTIC CHARITIES

	(7)
Risk Attitude	.004 (.009)
Ambiguity Aversion	-.011 (.009)
Numeracy	.019 (.038)
Empathy	-.002 (.004)
Optimism	-.007 (.005)
Donor Type	
Warm-Glow	.001 (.089)
Pure Altruism	-.031 (.102)
Age	.002 (.003)
Gender	.113 (.085)
Education	
Undergraduate degree	.021 (.088)
Postgraduate/Professional degree	.017 (.089)
Religion	
Protestantism	-.082 (.112)
Catholicism	-.076 (.107)
Islam	-.111 (.179)
Judaism	-
Buddhism	-
Hinduism	-
Religious Participation	.068 (.145)
Marriage Status	-.012 (.083)
Children	-.052 (.091)
Financial Wellbeing	-.012 (.040)
Employment	
Out of the workforce	-.028 (.170)
Part-time employment	.132 (.152)
Full-time employment	.097 (.141)
R^2	.084
Sample size	137

Notes: Unstandardised coefficients and standard errors. *p<.1, **p<.05, ***p<.01, ****p<.001
