# Supplementary material for: Sure-thing vs. probabilistic charitable giving: Experimental evidence on the role of individual differences in risky and ambiguous charitable decision-making
Source: PLoS One. 2022 Sep 22;17(9):e0273971. doi: 10.1371/journal.pone.0273971 (PMC9499298; doi:10.1371/journal.pone.0273971)
Supplement: S3 Appendix — (PDF) [file pone.0273971.s003.pdf]

Appendix C – Robustness Checks for Final Choice

In Appendix Table 5, Model (8), we provide a logit model for Model (2) to check for robustness of results. The results are the same as in Model (2).

APPENDIX TABLE 5—REGRESSION RESULTS FOR FINAL CHOICE – LOGIT ROBUSTNESS CHECK
PREDICTING FREQUENCY OF DONATION

	(8)
Risk Attitude	.009 (.022)
Ambiguity Aversion	-.010 (.020)
Numeracy	.098 (.087)
Empathy	.036**** (.009)
Optimism	.016 (.013)
Donor Type	
Warm-Glow	.268 (.225)
Pure Altruism	.230 (.278)
Condition X Risk Attitude	.016 (.032)
Condition X Ambiguity Aversion	.012 (.028)
Condition X Numeracy	.003 (.099)
Condition X Empathy	-.006 (.011)
Condition X Optimism	-.017 (.017)
Donor Type	
Condition X Warm-Glow	-.097 (.322)
Condition X Pure Altruism	.897** (.400)
Age	.003 (.006)
Gender	-.272* (.156)
Education	
Undergraduate degree	-.262 (.161)
Postgraduate/Professional degree	.158 (.173)
Religion	
Protestantism	.366 (.214)
Catholicism	.246 (.238)
Islam	.863** (.428)
Judaism	.673 (.683)
Buddhism	.710 (.753)
Hinduism	.712 (.624)
Sikhism	.264 (1.273)
Religious Participation	.071 (.264)
Marriage Status	-.120 (.161)
Children	.161 (.175)
Financial Wellbeing	.116 (.076)
Employment	
Out of the workforce	.083 (.284)
Part-time employment	-.106 (.250)

Full-time employment	-.156 (.221)
Cox and Snell R^2	.075
Sample size	1177

Notes: Log-odds and standard errors. *p<.1, **p<.05, ***p<.01, ****p<.001

In Appendix Table 6, we report the four pre-registered models for Final Choice. These did not include interactions but instead split the models by type of charity presented to them. Model (9) and Model (10) have frequency of donations to sure-thing and probabilistic charities as their outcome variables respectively. Model (11) and Model (12) are of the corresponding size of donation. These results are largely in line with Model (2) and Model (3), in that empathy is predictive of donation behaviour generally, but it is the pure altruistic donor type that best predict donation choices towards probabilistic charities in Final Choice.

APPENDIX TABLE 6—REGRESSION RESULTS FOR FINAL CHOICE – PRE-REGISTERED REGRESSION MODELS PREDICTION FREQUENCY AND SIZE OF DONATION

	(9)	(10)	(11)	(12)
Risk Attitude	.003 (.005)	.003 (.004)	.150 (.147)	.109 (.095)
Ambiguity Aversion	-.003 (.004)	<.001 (.003)	-.131 (.129)	-.019 (.077)
Numeracy	.033 (.023)	.008 (.017)	.598 (.653)	-.495 (.481)
Empathy	.008**** (.002)	.005** (.002)	.258**** (.06)	.082* (.043)
Optimism	.002 (.003)	.001 (.003)	.042 (.090)	.047 (.057)
Donor Type				
Warm-Glow	.047 (.051)	.036 (.040)	2.033 (1.485)	1.346 (.914)
Pure Altruism	.059 (.063)	.216**** (.056)	.315 (1.825)	2.806** (1.284)
Age	.001 (.002)	<.001 (.001)	.042 (.054)	.036 (.034)
Gender	-.038 (.087)	-.064* (.038)	-.255 (1.387)	-1.365 (.874)
Education				
Undergraduate degree	-.074 (.048)	-.057 (.044)	-2.564* 1.3741)	.079 (.907)
Postgraduate/Professional	.003 (.055)	.057 (.054)	-.757 (1.574)	.589 (1.007)
Religion				
Protestantism	.107 (.071)	.051 (.054)	.147 (2.042)	.589 (1.225)
Catholicism	.059 (.077)	.055 (.060)	3.103 (2.222)	.855 (1.361)
Islam	.286* (.158)	.106 (.106)	9.431** (4.550)	4.612* (2.404)
Judaism	.228 (.341)	.110 (.151)	6.908 (9.846)	2.308 (3.429)
Buddhism	.297 (.287)	.099 (.191)	15.139* (8.273)	.117 (4.338)
Hinduism	.226 (.216)	.080 (.154)	8.880 (6.223)	4.785 (3.507)
Sikhism	.199 (.343)	-.259 (.423)	-1.570 (9.899)	-5.135 (9.648)
Religious Participation	.015 (.090)	-.006 (.067)	.390 (2.592)	-1.723 (1.563)
Marriage Status	-.006 (.050)	-.039 (.040)	.387 (1.435)	-1.082 (.912)
Children	.074 (.053)	-.006 (.043)	.926 (1.534)	.737 (.978)
Financial Wellbeing	.038* (.023)	.007 (.019)	.962 (.655)	-.096 (.439)

Employment				
Out of the workforce	.093 (.092)	-.030 (.068)	1.320 (2.641)	-2.080 (1.550)
Part-time employment	.010 (.079)	-.043 (.059)	-1.060 (2.274)	-3.141** (1.353)
Full-time employment	-.014 (.069)	-.038 (.054)	.571 (1.982)	-2.985** (1.231)
R^2	.080	.054	.086	.052
Sample size	546	631	546	631

Notes: Unstandardised coefficients and standard errors. *p<.1, **p<.05, ***p<.01, ****p<.001

Models (13) and (14) in Appendix Table 7 present the results of Models (9) and (10) from Appendix Table 6 with logit models and otherwise identical specifications. We find no difference in results.

APPENDIX TABLE 7— REGRESSION RESULTS FOR FINAL CHOICE – LOGIT ROBUSTNESS CHECKS FOR PRE-REGISTERED MODELS PREDICTING FREQUENCY OF DONATION

	(13)	(14)
Risk Attitude	.015 (.023)	.017 (.024)
Ambiguity Aversion	-.014 (.021)	.003 (.020)
Numeracy	.158 (.106)	.049 (.103)
Empathy	.038**** (.011)	.029** (.012)
Optimism	.011 (.014)	.005 (.015)
Donor Type		
Warm-Glow	.215 (.234)	.223 (.235)
Pure Altruism	.274 (.286)	1.101**** (.294)
Age	.002 (.009)	.002 (.009)
Gender	-.189 (.221)	-.369* (.225)
Education		
Undergraduate degree	-.353 (.222)	-.199 (.243)
Postgraduate/Professional degree	.010 (.247)	.291 (.306)
Religion		
Protestantism	.476 (.313)	.291 (.306)
Catholicism	.275 (.346)	.321 (.338)
Islam	1.256* (.702)	.577 (.596)
Judaism	1.018 (1.467)	.552 (.782)
Buddhism	1.290 (1.297)	.452 (.989)
Hinduism	.978 (.955)	.451 (.879)
Sikhism	.888 (1.480)	-.20.097 (40192.970)
Religious Participation	.071 (.398)	-.015 (.377)
Marriage Status	-.029 (.227)	-.228 (.236)
Children	.343 (.245)	-.048 (.253)
Financial Wellbeing	.173* (.105)	.038 (.113)
Employment		
Out of the workforce	.431 (.422)	-.161 (.394)
Part-time employment	.073 (.370)	-.250 (.348)
Full-time employment	-.040 (.327)	-.212 (.311)

Cox and Snell R^2	.079	.051
Sample size	546	631

Notes: Log-odds and standard errors. *p<.1, **p<.05, ***p<.01, ****p<.001
