# Supplementary material for: Sure-thing vs. probabilistic charitable giving: Experimental evidence on the role of individual differences in risky and ambiguous charitable decision-making
Source: PLoS One. 2022 Sep 22;17(9):e0273971. doi: 10.1371/journal.pone.0273971 (PMC9499298; doi:10.1371/journal.pone.0273971)
Supplement: S4 Appendix — (PDF) [file pone.0273971.s004.pdf]

Appendix D – Additional Hypotheses Tests with Inconclusive Results

In this part of the appendix, we report the results regarding null hypotheses #3 and #4. Due to the low sample size, these analyses do not have the power we outlined above and are as such taken to be inconclusive. However, we still report all analyses as pre-registered.

First, we find that 83.3% of those who donate choose a sure-thing charity, and their average donation (M=53.91, SD=24.52) does not differ statistically significantly, t(40)=.818, p=.418, from those who donate to a probabilistic charity (M=45.71, SD=22.44). In Appendix Table 8, we report the results of the context-free condition, in which the design of Main Choice was mirrored but all identifying information and context was removed from the charity pair. Model (15) reports an OLS model and Model (16) tests the robustness of these results in a logit model. Both produce the same results. For both analyses, we excluded variables that had less than or equal to one instance of '1'. Given the low sample size we do not interpret these results with regard to null hypothesis #3.

APPENDIX TABLE 8—REGRESSION RESULTS FOR NO CONTEXT CONDITION – OLS AND LOGIT PREDICTING CHOICE BETWEEN SURE-THING AND PROBABILISTIC CHARITIES

	(15)	(16)
Risk Attitude	-.012 (.024)	-4.401 (913.808)
Ambiguity Aversion	.003 (.017)	.816 (1138.332)
Numeracy	.099 (.121)	89.731 (11034.812)
Empathy	.017 (.011)	4.576 (810.673)
Optimism	-.012 (.011)	-4.083 (635.613)
Donor Type		
Warm-Glow	-.043 (.223)	-9.676 (10508.857)
Pure Altruism	-.016 (.197)	40.576 (7375.105)
Age	.005 (.008)	4.758 (613.650)
Gender	.078 (.182)	54.477 (10072.358)
Education		
Undergraduate degree	.048 (.177)	64.545 (11881.462)
Postgraduate/Professional degree	-.196 (.224)	32.012 (19491.480)
Religion		
Protestantism	-.625 (.437)	-111.071 (26044.398)
Catholicism	-.101 (.286)	-108.735 (21698.451)
Islam	-	-
Judaism	-	-
Buddhism	-	-
Hinduism	-	-
Religious Participation	.080 (.362)	-72.564 (35338.129)
Marriage Status	-.056 (.189)	-92.326 (14588.695)
Children	-.068 (.221)	-6.219 (9909.734)

Financial Wellbeing	.017 (.100)	10.290 (4125.579)
Employment		
Out of the workforce	.293 (.491)	-48.198 (14982.568)
Part-time employment	-.110 (.259)	-92.719 (13479.830)
Full-time employment	.073 (.218)	-36.104 (8611.194)
R^2 and Cox & Snell R^2	.394	.594
Sample size	42	42

Notes: Unstandardized coefficients and standard errors (Model 1) as well as log-odds and standard errors (Model 2). *p<.1, **p<.05, ***p<.01, ****p<.001

Second, in Appendix Table 9, we test the effects of the informational treatment. We find that participant choices are not meaningfully different between the Main Choice, the expected-value treatment, and the made-up midontic theory treatment, see Appendix Table 9. The percentage point difference in the relative frequency of donations between the Main Condition and the expected-value treatment is 0.5p.p., whereas the difference between the Main Condition and the control is 0.6 p.p. Further, the direction of the change is contra the predicted directions, as the expected-value treatment seems to have resulted in a miniscule increase in donations to sure-thing charities. Given the low sample size we do not interpret these results with regard to null hypothesis #3.

APPENDIX TABLE 9—EXPECTED VALUE TREATMENT EFFECT

	Main Condition	Expected-Value Treatment	Midontic Theory Treatment
Sure-Thing Charity	84.7%	85.2%	84.1%
Probabilistic Charity	15.3%	14.8%	15.9%

Notes: Frequency of donations to sure-thing and probabilistic charities.
