# Supplementary material for: Sure-thing vs. probabilistic charitable giving: Experimental evidence on the role of individual differences in risky and ambiguous charitable decision-making
Source: PLoS One. 2022 Sep 22;17(9):e0273971. doi: 10.1371/journal.pone.0273971 (PMC9499298; doi:10.1371/journal.pone.0273971)
Supplement: S5 Appendix — (PDF) [file pone.0273971.s005.pdf]

Appendix E – Additional Equivalence Tests

In Appendix Table 10, we report an additional series of equivalence tests for Final Choice. In this series of tests, we focus on Table 4, Model (3) and the size of donation. As before, because we did not pre-register equivalence bounds we report a number of them to check for sensitivity of results to choice of equivalence bounds. Note though that in contrast to previous equivalence tests, this set of equivalence bounds is defined with regard to standardised regression coefficients from Model (3). Here we find that at the equivalence bounds of (-).25, risk attitudes, ambiguity aversion, and donor type are negligibly small. At the (-).5 level, numeracy and optimism are also negligibly small within these modestly large parameters. We do not have sufficient evidence in favour of a negligibly small effect even at (-).5 for empathy. We thus argue that we have relatively strong evidence in favour of risk attitudes, ambiguity aversion, and donor type as being negligibly small, while the other variables provide less strong evidence in favour of this.

APPENDIX TABLE 10—TOST FOR FINAL CHOICE COEFFICIENTS

	-.1	.1	-.25	.25	-.5	.5
Condition X Risk Attitudes	1.61*	1.37*	3.85****	3.61****	7.58****	7.34****
Condition X Ambiguity Aversion	2.86***	1.68**	6.27****	5.09****	11.96****	10.77****
Condition X Numeracy	1.10	-.04	1.89**	.23	3.22****	2.07**
Condition X Empathy	-0.99	2.09**	-.16	2.92**	1.22	4.30****
Condition X Optimism	.40	1.16	1.58*	2.33**	3.53****	4.28****
Condition X Warm Glow	1.53*	2.72**	4.72****	5.91****	10.04****	11.23****
Condition X Pure Altruism	1.02	3.74****	4.56****	7.31****	10.55****	13.26****

Notes: All t-test results for TOST procedures on a variety of lower and upper equivalence bounds (in standardized coefficients) from Model (3). *p<.1, **p<.05, ***p<.01, ****p<.001
