# Supplementary material for: Sure-thing vs. probabilistic charitable giving: Experimental evidence on the role of individual differences in risky and ambiguous charitable decision-making
Source: PLoS One. 2022 Sep 22;17(9):e0273971. doi: 10.1371/journal.pone.0273971 (PMC9499298; doi:10.1371/journal.pone.0273971)
Supplement: S6 Appendix — (PDF) [file pone.0273971.s006.pdf]

Appendix F – Additional Regression for Main Choice

In Appendix Table 11, Model (17), we report the same OLS regression for Main Choice as in Model (1). The main change in this regression is that, in response to the worry that by excluding those that do not donate, we may be biasing our estimates as participants may be indifferent between donating a non-zero amount to one type of charity (say a sure-thing charity) and no donation to a probabilistic charity. As we had additional data on all participants from their choices in Final Choice, we excluded the following participants on top of our standard exclusion criteria. First, we excluded those who donated to a sure-thing charity in Main Choice but did not donate at all in Final Choice when they were shown a probabilistic charity (n=70). Second, we also excluded those who donated to a probabilistic charity in Main Choice but who did not donate at all in Final Choice when they were shown a sure-thing charity (n=5). Overall, we do not find a difference in results which suggests that that our data may go some way towards addressing this worry.

APPENDIX TABLE 11—REGRESSION RESULTS FOR CHARITABLE GIVING BEHAVIOUR IN MAIN CHOICE
PREDICTING CHOICE BETWEEN SURE-THING AND PROBABILISTIC CHARITIES

	(17)
Risk Attitude	.000 (.007)
Ambiguity Aversion	-.004 (.006)
Numeracy	-.005 (.030)
Empathy	-.001 (.003)
Optimism	-.004 (.004)
Donor Type	
Warm-Glow	-.012 (.065)
Pure Altruism	-.086 (.076)
Donation (amount)	-.001 (.001)
Age	.004 (.002)
Gender	-.002 (.062)
Education	
Undergraduate degree	.068 (.064)
Postgraduate/Professional degree	-.017 (.069)
Religion	
Protestantism	-.116 (.084)
Catholicism	-.045 (.089)
Islam	-.111 (.171)
Judaism	-.198 (.412)
Buddhism	-.151 (.318)
Hinduism	.319 (.303)
Religious Participation	.057 (.109)

Marriage Status	.044 (.062)
Children	.000 (.069)
Financial Wellbeing	.007 (.030)
Employment	
Out of the workforce	-.213 (.122)
Part-time employment	-.005 (.104)
Full-time employment	-.099 (.096)
R^2	.081
Sample size	232

Notes: OLS regression reporting unstandardised coefficients and standard errors. Outcome variable is charity choice (0 = sure-thing charity, 1 = probabilistic charity). *p<.1, **p<.05, ***p<.01, ****p<.001
