# Supplementary material for: Sure-thing vs. probabilistic charitable giving: Experimental evidence on the role of individual differences in risky and ambiguous charitable decision-making
Source: PLoS One. 2022 Sep 22;17(9):e0273971. doi: 10.1371/journal.pone.0273971 (PMC9499298; doi:10.1371/journal.pone.0273971)
Supplement: S7 Appendix — (PDF) [file pone.0273971.s007.pdf]

Appendix G – Experimental Texts

Below we show the main experimental texts. See Supplemental Materials for full experimental procedure including all instructions.

Charity Descriptions

SCI Foundation (Sure-Thing Charity #1)
The SCI Foundation focuses on improving global health by combating parasitic worm infections. It works with governments or non-governmental actors worldwide to treat a number of important diseases. For example, these diseases include schistosomiasis and soil-transmitted helminthiasis, both commonly known as 'worm infections.' These infections are treated by providing deworming drugs once a year that significantly reduce the worm load and cure the present infection.

According to recent trials, multiple dose interventions have a cure-rate of 96.1%. The total cost of deworming is about $1.03. Previous research found that deworming campaigns improve the health of the treated children (increasing weight and haemoglobin levels), prevent potentially severe outcomes (such as organ damage), as well as reduce school absenteeism, increase cognitive function, and show an improvement in labour force participation and incomes in both the treated population and the community as a whole.

Donating to the SCI Foundation contributes to this deworming effort and will reliably improve the lives of many as well as their communities. There is a yearly shortfall of funding, and donating will directly help those in need.

GiveDirectly (Sure-Thing Charity #2)
GiveDirectly has as its goal to directly send cash to people in poverty in order to allow those living in extreme poverty to spend cash on whatever it is they need most. It works with governments or non-governmental actors worldwide to address extreme poverty. These cash transfers only reach people who are living in extreme poverty and are suffering from hunger, a lack of shelter, and easily preventable diseases. Cash transfers can help the recipients alleviate these conditions.

In 2017, 90% of recipients received the payments within 70 days. For every $1 donated, GiveDirectly delivers $0.83 directly to those in need. Recent research investigating what recipients actually spend their transfers on found that it was spent on livestock, durable goods, savings, business expenses, health expenditures, education expenditures, and consumption overall (though alcohol and tobacco spending did not increase), overall increasing their welfare and the welfare of their communities.

Donating to GiveDirectly contributes to this poverty alleviation effort and will reliably improve the lives of many as well as their communities. There is a yearly shortfall of funding, and donating will directly help those in need.

Against Malaria Foundation (Sure-Thing Charity #3)
The Against Malaria Foundation has as its goal to directly distribute long-lasting insecticide-treated mosquito nets in at-risk low-income communities that stop the transmission of malaria. It works with governments or non-governmental actors worldwide to address this issue of malaria deaths. Providing mosquito nets can help the recipients reduce the chance of falling ill with malaria.

In 2019, a follow-up monitoring conducted between 6 and 23 months showed that 90% of recipients that received a net had applied it properly. The cost to purchase and distribute such an insecticide treated net is $4.95. This means that providing insecticide-treated bed nets is an effective way of reducing malaria-related deaths and thus improving the welfare of recipients and their communities.

Donating to the Against Malaria Foundation contributes to the overall effort at reducing the impact of malaria and will reliably improve the lives of many as well as their communities. There is a yearly shortfall of funding, and donating will directly help those in need.

Machine Intelligence Research Institute (Probabilistic Charity #1)
The Machine Intelligence Research Institute has as its goal to prevent or reduce the risk of artificial intelligence/machine intelligence threats. It works with governments or non-governmental actors worldwide to prepare for the potential threat that intelligent systems pose to humanity. In their work they aim to research and make safer the underlying principles and applications of artificial intelligence.

According to a recent estimation, there is a 5% risk over the next century that artificial intelligence may pose a risk to humans at the scale of human extinction (billions of lives lost). The Machine Intelligence Research Institute works towards laying the foundations for the safety of future intelligence systems to better understand the implications of such technological advances. This work promises to help reduce the threat of these rare outcomes of intelligent systems and minimise the risk that humanity is exposed to every year. The charity's interventions work to reduce this chance by an unknown probability.

Donating to the Machine Intelligence Research Institute will directly help prepare humanity for those very rare but important technological risks by reducing the chance that any such technology will end up having a highly destructive impact. This can be achieved by developing technological solutions that may make such catastrophes less likely. Donations are needed every year to have a chance at making an impact in this area.

The Center for Health Security (Probabilistic Charity #2)
The Center for Health Security has as its mission the aim of protecting global health by focusing on epidemics and disasters and by ensuring that communities are resilient to major health challenges. It works with governments or non-governmental actors worldwide to prepare for future health disasters like emerging infectious diseases. For example, they run the 'Outbreak Observatory' in which outbreaks of new diseases are monitored and which contributes to preventing and responding to these outbreaks.

Recent estimates show that the yearly chance of a severe flu outbreak is between 0.5 and 1%. Such an influenza pandemic of these proportions could result in a 5% reduction in global GDP as well as between 21 and 33 million deaths worldwide. Preparing for such rare events in advance might make them less likely to happen or at least less devastating. The charity's interventions work to reduce this chance by an unknown probability.

Donating to the Center for Health Security will help prepare humanity for those very rare but important health risks by reducing the chance of such an outbreak as well as the potential severity of it. This can be achieved by being quicker at identifying or combating novel outbreaks. Donations are needed every year to have a chance at making an impact in this area.

Nuclear Threat Initiative (Probabilistic Charity #3)
The Nuclear Threat Initiative has as its goal to prevent catastrophic attacks with weapons of mass destruction such as nuclear weapons. It works with governments or non-governmental actors worldwide to prepare for the threat of nuclear war. In their work they aim to strengthen non-proliferation and disarmament agreements and advance international partnerships for nuclear disarmament verification.

According to a survey of experts, the chance of a nuclear war that kills at least 1 million people is about 0.39% every year. It has also been estimated that between 30 and 75 million people would die worldwide as a result of such a nuclear war between the US and Russia. Preparing for such rare events in advance might make them less likely to happen or at least less devastating. The charity's interventions work to reduce this chance by an unknown probability.

Donating to the Nuclear Threat Initiative will help prepare humanity for those very rare but important war risks by reducing the chance of such a conflict, as well as the potential severity of it. This can be achieved by forming new treaties or by fostering international collaboration. Donations are needed every year to have a chance at making an impact in this area.

Context-free charity texts

Charity 1 (Context-free sure-thing charity)
This charity focuses on helping people in need. Its interventions have a success rate of 96.1%. The charity's interventions improve the well-being of those treated and lead to a number of additional positive consequences. Donating to this charity reliably improves lives of many as well as their communities. For this charity, there is a yearly shortfall of funding to achieve this goal.

Charity 2 (Context-free probabilistic charity)
This charity focuses on preventing global catastrophes. These catastrophes have a chance of 0.39% to happen every year, and if they happened might cost the lives of between 30 and 75 million people worldwide. The charity's interventions work to reduce this chance by an unknown probability. Donating to this charity might make these catastrophes less likely to happen or at least less devastating. For this charity, there is a yearly shortfall of funding to achieve this goal.

Expected value treatment text (left) and control (right)

Before you continue in this survey, we would like to introduce you to a theory of decision-making.

We must often make decisions under conditions of uncertainty. One theory of how to best make those decisions is called Expected Value Theory. Its basic slogan is: Choose the act with the highest expected value.

The expected value of an act is a weighted average of the values of each of its possible outcomes. The value of each outcome is weighted according to the probability that the act will lead to that outcome. Consider the following example:

A lottery ticket costs $5. The probability of winning the $2000 prize is 0.5% The likely value from having a lottery ticket will be the outcome multiplied by the probability of the event occurring. Therefore, expected value = 0.005 x 2000 = $10.

The expected value of owning a lottery ticket is $10. The expected value is the average of your payouts if you were to make the same decision many times. Of course, if you make the decision only once, your payout will be the value of the outcome the lottery, but before the lottery is played what you expect to earn is its expected value, and thus it pays off to follow expected value reasoning. Since the ticket costs $5, according to the expected value calculations, buying the ticket is a good decision – because the expected value of buying a ticket is $10, which is higher than the cost of purchase ($5).

As such, expected value reasoning can sometimes allow one to see that betting on low probability outcomes can be beneficial for as long as the outcomes are good enough.

Before you continue in this survey, we would like to introduce you to a theory of decision-making.

We must often make decisions under conditions of uncertainty. One theory of how to best make those decisions is called Midontic Choice Theory. Its basic slogan is: Choose the act with the highest midontic choice value.

The midontic choice value of an act is a function of how unlikely an outcome is. This is calculated by taking the value of the least likely outcome and multiplying it by the midontic parameter. This midontic parameter is calculated by taking the value of the most likely outcome and adding 0.5 to it. Consider the following example:

A lottery ticket costs $5. The probability of winning the $2000 prize is 0.5%. The midontic parameter for this lottery is 0+0.5=0.5. In order to calculate the midontic choice value, one has to then multiply this parameter by the value of the least likely outcome ($2000): 0.5 x 2000 = $1000.

The midontic choice value of owning a lottery ticket for this lottery is $1000. Since the ticket costs $5, according to midontic value calculations, buying the ticket is a good decision – because the midontic value of buying a ticket is $1000, which is higher than the cost of purchase ($5).

As such, midontic value reasoning can sometimes allow one to see that betting on low probability outcomes can be beneficial for as long as the outcomes are good enough.
