# Supplementary material for: Sure-thing vs. probabilistic charitable giving: Experimental evidence on the role of individual differences in risky and ambiguous charitable decision-making
Source: PLoS One. 2022 Sep 22;17(9):e0273971. doi: 10.1371/journal.pone.0273971 (PMC9499298; doi:10.1371/journal.pone.0273971)
Supplement: S1 File — (PDF) [file pone.0273971.s008.pdf]

Welcome to this study! Throughout this study you will be asked to finish several short surveys and tasks. For example, these might include surveys about yourself or tasks in which you can earn additional money. We ask you to read the descriptions of all tasks carefully and take your time with them before you make your decisions.

You will be paid **£2.00 for participation** in this study that is yours to keep. Participation should take between 10 and 15 minutes. On top of this, you are able to **earn up to £1.83 based on your choices** in this survey. All of that money that you do not allocate during the survey will be paid out to you directly via Prolific. Please note that the study is denominated in GBP (British pounds) with £1 = 100 pence.

To make sure that we can pay out your additional earnings correctly and in a timely manner, please enter your Prolific ID. If you do not enter this ID, we might not be able to pay out any additional earnings.

>>

We will only collect data on your choices within this study. All data is anonymised. We only collect your Prolific ID so we can pay out the bonus at the end of study.

Your participation in this study is entirely voluntary and you can withdraw at any point should you wish to do so. You are also free to omit answering any question. We believe that there are no risks associated with this study; however, as with any online related activity the risk of a breach is always possible. This is why all answers will be fully anonymised.

If you would like to participate in this study, please click the "I consent" button below. If you do not wish to do so, please click the "I do not consent" button below.

- ◯ I consent.
- ◯ I do not consent.

>>

In this first part of the survey, you will be asked to fill out three questionnaires. **Each questionnaire that you have filled out will reward you an additional £0.25 (25 pence).**

>>

Please take your time and answer these questions carefully.

Imagine that we roll a fair, six-sided die 1000 times. Out of 1000 rolls, how many times do you think the die would come up as an even number?

- ◯ 50 out of 1000
- ◯ 500 out of 1000
- ◯ 750 out of 1000

In the BIG BUCKS LOTTERY, the chances of winning a $10.00 prize are 1%. What is your best guess about how many people would win a $10.00 prize if 1000 people each buy a single ticket from BIG BUCKS?

- ◯ 10 persons out of 1000
- ◯ 100 persons out of 1000
- ◯ 200 persons out of 1000

In the ACME PUBLISHING SWEEPSTAKES, the chance of winning a car is 1 in 1000. What percent of tickets of ACME PUBLISHING SWEEPSTAKES win a car?

- ◯ 0.01%
- ◯ 0.1%
- ◯ 1%

Which of the following numbers represents the biggest risk of getting a disease?

- ◯ 1 in 100
- ◯ 1 in 1000
- ◯ 1 in 10

Which of the following numbers represents the biggest risk of getting a disease?

- ◯ 1%
- ◯ 5%
- ◯ 10%

If Person A's risk of getting a disease is 1% in 10 years, and Person B's risk is double that of A's, what is B's risk?

- 0.1%
- 1%
- 2%

If Person A’s chance of getting a disease is 1 in 100 in ten years, and person B’s risk is double that of A’s, what is B’s risk?

- 0.5 out of 100
- 1 out of 100
- 2 out of 100

If the chance of getting a disease is 10%, how many people would be expected to get the disease out of 100?

- 1 person
- 10 people
- 100 people

If the chance of getting a disease is 20 out of 100, this would be the same as having a _____% chance of getting the disease.

- 10%
- 20%
- 40%

The chance of getting a viral infection is .0005. Out of 10,000 people, about how many of them are expected to get infected?

- 5 people
- 50 people
- 500 people

>>

Please take your time and answer these questions carefully.

Please indicate on a scale from 'I disagree a lot' to 'I agree a lot' your (dis)agreement with the following statements.

	I disagree a lot	I disagree a little	I neither agree nor disagree	I agree a little	I agree a lot
In uncertain times, I usually expect the best.	○	○	○	○	○
It's easy for me to relax.	○	○	○	○	○
If something can go wrong for me, it will.	○	○	○	○	○
I'm always optimistic about my future.	○	○	○	○	○
I enjoy my friends a lot.	○	○	○	○	○
It's important for me to keep busy	○	○	○	○	○
I hardly ever expect things to go my way.	○	○	○	○	○
I don't get upset too easily.	○	○	○	○	○
I rarely count on good things happening to me.	○	○	○	○	○
Overall, I expect more good things to happen to me than bad.	○	○	○	○	○
It is important that you pay attention to this study. Please tick 'I agree a little'.	○	○	○	○	○

>>

Please indicate on a scale from 'Strongly Disagree' to 'Strongly Agree' your (dis)agreement with the following statements.

	Strongly Disagree	Disagree	Neither Agree nor Disagree	Agree	Strongly Agree
My friends' emotions don't affect me much.	○	○	○	○	○
After being with a friend who is sad about something, I usually feel sad.	○	○	○	○	○
I can understand my friend's happiness when she/he does well at something.	○	○	○	○	○
I get frightened when I watch characters in a good scary movie.	○	○	○	○	○
I get caught up in other people's feelings easily.	○	○	○	○	○
I find it hard to know when my friends are frightened.	○	○	○	○	○
I don't become sad when I see other people crying.	○	○	○	○	○
Other people's feeling don't bother me at all.	○	○	○	○	○
When someone is feeling 'down' I can usually understand how they feel.	○	○	○	○	○
I can usually work out when my friends are scared.	○	○	○	○	○
I often become sad when watching sad things on TV or in films.	○	○	○	○	○
I can often understand how people are feeling even before they tell me.	○	○	○	○	○
Seeing a person who has been angered has no effect on my feelings.	○	○	○	○	○
I can usually work out when people are cheerful.	○	○	○	○	○
I tend to feel scared when I am with friends who are afraid.	○	○	○	○	○
I can usually realize quickly when a friend is angry.	○	○	○	○	○
I often get swept up in my friends' feelings.	○	○	○	○	○
My friend's unhappiness doesn't make me feel anything.	○	○	○	○	○
I am not usually aware of my friends' feelings.	○	○	○	○	○
I have trouble figuring out when my friends are happy.	○	○	○	○	○

>>

Thank you for completing the first part of this survey. **Completing this has earned you £0.75 (75 pence).**

Before continuing, **you now have the option to donate all, some, or none of your earned £0.75 to one of two charities below.** Both charities have been selected by Giving What We Can, a charity evaluation company, as **top charities to donate to in 2021.**

However much you end up not donating here will be paid out directly to you after the survey via Prolific.

>>

Below please find two charities that you can decide to donate to.

SCI Foundation
The SCI Foundation focuses on improving global health by combating parasitic worm infections. It works with governments or non-governmental actors worldwide to treat a number of important diseases. For example, these diseases include schistosomiasis and soil-transmitted helminthiasis, both commonly known as 'worm infections.' These infections are treated by providing deworming drugs once a year that significantly reduce the worm load and cure the present infection.

According to recent trials, multiple dose interventions have a cure-rate of 96.1%. The total cost of deworming is about $1.03. Previous research found that deworming campaigns improve the health of the treated children (increasing weight and haemoglobin levels), prevent potentially severe outcomes (such as organ damage), as well as reduce school absenteeism, increase cognitive function, and show an improvement in labour force participation and incomes in both the treated population and the community as a whole.

Donating to the SCI Foundation contributes to this deworming effort and will reliably improve the lives of many as well as their communities. There is a yearly shortfall of funding, and donating will directly help those in need.

The Center for Health Security
The Center for Health Security has as its mission the aim of protecting global health by focusing on epidemics and disasters and by ensuring that communities are resilient to major health challenges. It works with governments or non-governmental actors worldwide to prepare for future health disasters like emerging infectious diseases. For example, they run the 'Outbreak Observatory' in which outbreaks of new diseases are monitored and which contributes to preventing and responding to these outbreaks.

Recent estimates show that the yearly chance of a severe flu outbreak is between 0.5 and 1%. Such an influenza pandemic of these proportions could result in a 5% reduction in global GDP as well as between 21 and 33 million deaths worldwide. Preparing for such rare events in advance might make them less likely to happen or at least less devastating. The charity's interventions work to reduce this chance by an unknown probability.

Donating to the Center for Health Security will help prepare humanity for those very rare but important health risks by reducing the chance of such an outbreak as well as the potential severity of it. This can be achieved by being quicker at identifying or combating novel outbreaks. Donations are needed every year to have a chance at making an impact in this area.

You can now decide to donate to either of these charities or decide not to donate.

- ○ SCI Foundation.
- ○ The Center for Health Security.
- ○ I do not want to donate.

>>

In a previous part of this survey you were presented with two charities. We would now like you to evaluate these charities based on how impactful you think they are. Below you will see both charity texts again.

SCI Foundation

The SCI Foundation focuses on improving global health by combating parasitic worm infections. It works with governments or non-governmental actors worldwide to treat a number of important diseases. For example, these diseases include schistosomiasis and soil-transmitted helminthiasis, both commonly known as 'worm infections.' These infections are treated by providing deworming drugs once a year that significantly reduce the worm load and cure the present infection.

According to recent trials, multiple dose interventions have a cure-rate of 96.1%. The total cost of deworming is about $1.03. Previous research found that deworming campaigns improve the health of the treated children (increasing weight and haemoglobin levels), prevent potentially severe outcomes (such as organ damage), as well as reduce school absenteeism, increase cognitive function, and show an improvement in labour force participation and incomes in both the treated population and the community as a whole.

Donating to the SCI Foundation contributes to this deworming effort and will reliably improve the lives of many as well as their communities. There is a yearly shortfall of funding, and donating will directly help those in need.

The Center for Health Security

The Center for Health Security has as its mission the aim of protecting global health by focusing on epidemics and disasters and by ensuring that communities are resilient to major health challenges. It works with governments or non-governmental actors worldwide to prepare for future health disasters like emerging infectious diseases. For example, they run the 'Outbreak Observatory' in which outbreaks of new diseases are monitored and which contributes to preventing and responding to these outbreaks.

Recent estimates show that the yearly chance of a severe flu outbreak is between 0.5 and 1%. Such an influenza pandemic of these proportions could result in a 5% reduction in global GDP as well as between 21 and 33 million deaths worldwide. Preparing for such rare events in advance might make them less likely to happen or at least less devastating. The charity's interventions work to reduce this chance by an unknown probability.

Donating to the Center for Health Security will help prepare humanity for those very rare but important health risks by reducing the chance of such an outbreak as well as the potential severity of it. This can be achieved by being quicker at identifying or combating novel outbreaks. Donations are needed every year to have a chance at making an impact in this area.

Please indicate how impactful you think these two charities are. 0 = not impactful at all, 100 = extremely impactful.

Not impactful at all										Extremely impactful
0	10	20	30	40	50	60	70	80	90	100

SCI Foundation

The Center for Health Security

Please indicate how you anticipate others to evaluate these charities' impact. If you think that, on average, others will think that a given charity is extremely impactful, choose '100', if you think that, on average, others will think that a given charity is not impactful at all, 'choose 0' and so on.

After the survey, if your estimations are within 5 percentage points of the actual average, you will receive **an additional £0.10.**

Not impactful at all										Extremely impactful
0	10	20	30	40	50	60	70	80	90	100

SCI Foundation

The Center for Health Security

>>

In this last part of the survey **you will be able to earn up to £0.98 (98 pence) in addition to what you have earned before.** After that, you will be able to donate all, some, or none of that to charity. We ask you to indicate upfront how much you will donate given a certain amount of earnings below.

You can now indicate how much you will donate to the following charity.

Nuclear Threat Initiative
The Nuclear Threat Initiative has as its goal to prevent catastrophic attacks with weapons of mass destruction such as nuclear weapons. It works with governments or non-governmental actors worldwide to prepare for the threat of nuclear war. In their work they aim to strengthen non-proliferation and disarmament agreements and advance international partnerships for nuclear disarmament verification.

According to a survey of experts, the chance of a nuclear war that kills at least 1 million people is about 0.39% every year. It has also been estimated that between 30 and 75 million people would die worldwide as a result of such a nuclear war between the US and Russia. Preparing for such rare events in advance might make them less likely to happen or at least less devastating. The charity's interventions work to reduce this chance by an unknown probability.

Donating to the Nuclear Threat Initiative will help prepare humanity for those very rare but important war risks by reducing the chance of such a conflict, as well as the potential severity of it. This can be achieved by forming new treaties or by fostering international collaboration. Donations are needed every year to have a chance at making an impact in this area.

How much will you donate if you earn 14 pence? Please enter a number in pence.

How much will you donate if you earn 28 pence? Please enter a number in pence.

How much will you donate if you earn 42 pence? Please enter a number in pence.

How much will you donate if you earn 56 pence? Please enter a number in pence.

How much will you donate if you earn 70 pence? Please enter a number in pence.

How much will you donate if you earn 84 pence? Please enter a number in pence.

How much will you donate if you earn 98 pence? Please enter a number in pence.

>>

This last part of the study has two parts.

In this part, you will be able to earn **up to £0.48 (48 pence) in addition to what you have earned before.**

In this task, you will make one training decision and one actual decision. For each round, you will see a 5x5 grid containing 25 boxes, an example of which is shown below.

1	2	3	4	5
6	7	8	9	10
11	12	13	14	15
16	17	18	19	20
21	22	23	24	25

Most of these boxes are empty, but 1 of them contains a “bomb.” The placement of the bomb is completely random – each of the 25 boxes is equally likely to contain the bomb.

You can choose to open as many boxes as you like, from 0-25 (Note: This means you are not required to open any boxes if you do not wish to do so). **If you open the box that contains the bomb, you will not gain anything. However, if you only open empty boxes, you will gain £0.02 per box opened.**

You will click on all the boxes you wish to open, and then progress to the next page to reveal which box contained the bomb. You will NOT see whether a box contains the bomb until you’ve made ALL your choices on which boxes to open and have progressed to the next page. That is, if you've clicked on the bomb, you will not learn that immediately. You will see the bomb placement after you've opened all of the boxes you want to open and you progress to the next page.

You will make two decisions, one is purely for training so you are familiar with the procedure, and the second will actually affect your earnings.

>>

Before you begin, we will ask you to answer a few questions to test your understanding of the instructions.
Let's assume you open 12 boxes -- # 1, 3, 5, 7, 9, 11, 13, 15, 17, 19, 21, and 23. The bomb was behind box 2. How much would you earn in this round?

- ◯ £0.12
- ◯ £0.24
- ◯ £0.00

Again, let's assume you open boxes # 1, 3, 5, 7, 9, 11, 13, 15, 17, 19, 21, and 23. This time, the bomb was behind box 1. How much would you earn in this round?

- ◯ £0.00
- ◯ £0.12
- ◯ £0.14

This time, let's assume you open ALL 25 boxes. How much would you earn in this round?

- ◯ £0.00
- ◯ £0.40
- ◯ £0.14

>>

Great! You answered all of the questions correctly.

The more boxes you open, the more likely it is that you will open one of the boxes containing a bomb. But how likely is it, exactly?

Below, you'll find the mathematical likelihood of selecting a bomb, depending on how many total boxes you open.

If you open ______ total boxes...	There is a _____ chance you'll select a bomb	You would earn _____ if you do not select a bomb	You would earn _____ if you do select a bomb
0	0%	£0.00	N/A
1	4%	£0.02	£0.00
2	8%	£0.04	£0.00
3	12%	£0.06	£0.00
4	16%	£0.08	£0.00
5	20%	£0.10	£0.00
6	24%	£0.12	£0.00
7	28%	£0.14	£0.00
8	32%	£0.16	£0.00
9	36%	£0.18	£0.00
10	40%	£0.20	£0.00
11	44%	£0.22	£0.00
12	48%	£0.24	£0.00
13	52%	£0.26	£0.00
14	56%	£0.28	£0.00
15	60%	£0.30	£0.00
16	64%	£0.32	£0.00
17	68%	£0.34	£0.00
18	72%	£0.36	£0.00
19	76%	£0.38	£0.00
20	80%	£0.40	£0.00
21	84%	£0.42	£0.00
22	88%	£0.44	£0.00
23	92%	£0.46	£0.00
24	96%	£0.48	£0.00
25	100%	N/A	£0.00

For example, in opening 2 boxes you have an 8% chance of selecting a bomb. This means that if 100 people participate in this task and they each open 2 boxes, on average 8 of those 100 people will open a box containing a bomb, and they would earn £0.00. The remaining 92 people will open only empty boxes and these people would earn £0.04.

We will provide this table for you for reference, if you wish, as you make your decisions.

>>

This is just a practice round so you can see how it works when you click on the boxes. Go ahead and practice, clicking on as many boxes as you would like to open. You'll find out on the next screen whether you selected a bomb or not, and how much you would have earned if this were a real round.

Please remember that, if you click on a bomb, you won't be notified of that in "real-time." You'll find out after you have made all your choices and progressed to the next round.

1	2	3	4	5
6	7	8	9	10
11	12	13	14	15
16	17	18	19	20
21	22	23	24	25

>>

Click to reveal the bomb!

>>

The bomb was behind box 5. You did not select the bomb! This is just a practice round, so you won't actually earn anything from this round. But if it had been a real round, you would have earned 0 pence.

1	2	3	4	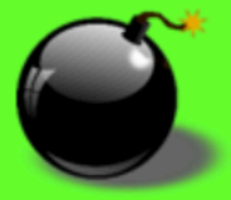
6	7	8	9	10
11	12	13	14	15
16	17	18	19	20
21	22	23	24	25

>>

You will now begin the main decision round.

Click on any number of boxes to open them. If the boxes you open do not contain a bomb, you will receive £0.02 for each one you open. If you do open a box containing a bomb, you will not gain any money.

1	2	3	4	5
6	7	8	9	10
11	12	13	14	15
16	17	18	19	20
21	22	23	24	25

>>

Click to reveal the bomb!

>>

You chose 2, 3, 4, 7, 8, 9.

The bomb was behind box 4. **You will not gain anything (£0.00).**

1	2	3	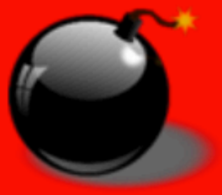	5
6	7	8	9	10
11	12	13	14	15
16	17	18	19	20
21	22	23	24	25

>>

Welcome to the second part of this section of the study. Here, you will be able to earn **up to £0.50 (50 pence) in addition to what you have earned before.**

In this section you can place a bet on the color of the ball drawn from each of two boxes. **If your bet on a specific box is correct, you will win £0.25. If your bet is incorrect, you earn nothing.**

For example, if both of your bets are correct, you win a total of £0.50. If neither are correct you win a total of £0.00.

You will face the following scenario: **there are two boxes, each containing 10 balls, which can be either red or black.** The number of balls in both boxes is as follows:

BOX 1: Contains 5 red balls and 5 black balls.

BOX 2: The number of red and black balls is unknown. It could be any number between 0 red balls (and 10 black balls) and 10 red balls (and 0 black balls).

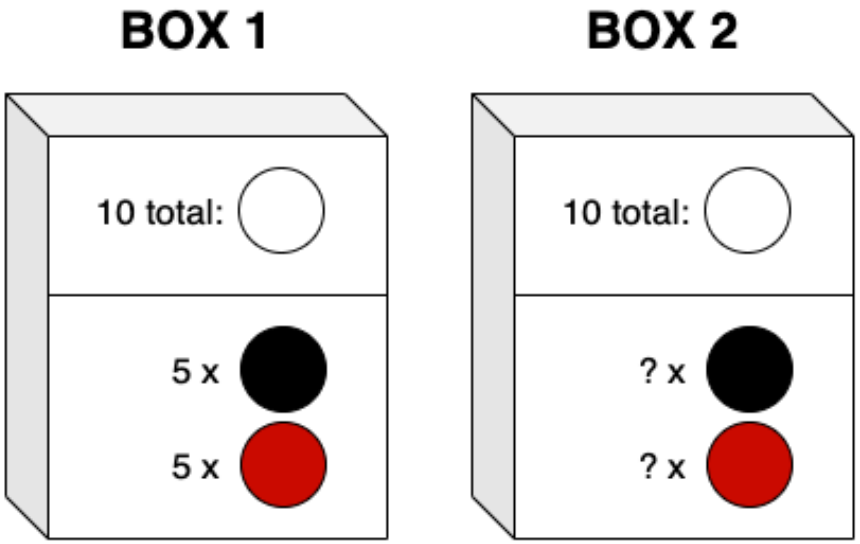

In this part, you will decide on which of the two colors (black or red) you want to bet on.

However, **before balls are drawn from each box, you may sell each one of your bets.** You are asked to state two **minimal prices at which you are willing to sell** each one of the bets. For each box, a random number between £0 and £0.25 will be generated. The random number is the buying price for the bet associated with the box.

If the buying price for a box is higher than the minimal selling price you stated for that box, you will be paid the buying price (and the outcome of the box will not determine your earnings). However, **if the buying price for the box is lower than the minimal selling price you stated for that box, your payment will depend on the outcome of your bet.**

Note that it is in your best interest not to overstate your selling price because this lowers the chances that you will be able to sell your bet and does not increase the buying price, which is drawn randomly, as explained above.

Likewise, it is in your best interest not to understate your selling price, because this may force you to sell a bet at a price that is lower than your valuation of the bet.

For example, suppose you want to sell a £1 coin you have. Clearly its value is exactly £1. If you state a selling price higher than £1 (say £1.50), you might not be able to sell it even if the buying price is as high as your selling price minus 1p (in the example, £1.49) - which would be a profitable transaction for any buying price above £1. Likewise, if you state a selling price lower than £1 (say £0.75), you might be forced to sell your coin at a loss (if the buying price is between £0.75 and £0.99). The only way you are sure not to lose is if you state a selling price of exactly your valuation (£1 in this case).

It is important that your stated selling prices reflect how attractive each bet is: The more attractive it is for you to participate in a bet, the higher the selling price you should state.

>>

Before we continue, we want to make sure you understand the mechanism.

Imagine you want to sell a pen and state a selling price of 100 pence. The random number that determines the buying price is drawn and is 110 pence.

Will you automatically sell your pen for 100 pence or will you keep your pen?

- (selected) The pen will automatically be sold for 110 pence (£1.10).
- ( ) The pen will not be automatically sold.

>>

At this stage you can now place your bets and state your selling prices for both of these bets. Remember that your choices are about the following two boxes (BOX 1 and BOX 2).

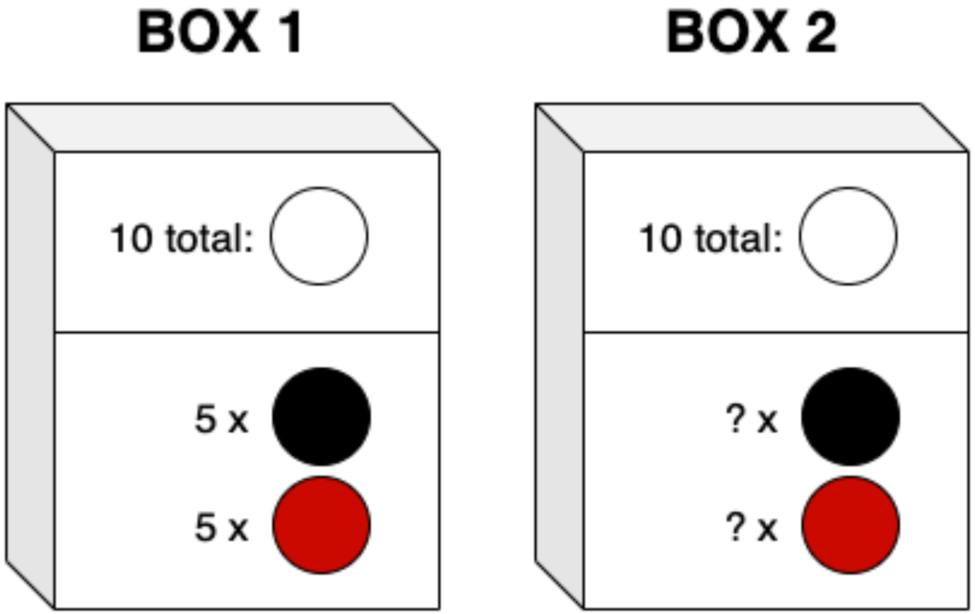

What is your bet for BOX 1? Remember that when you win your bet you will win 25 pence, but if you lose your bet you will not win anything.

- ○ Red
- ○ Black

What is your minimal reservation price for the bet on BOX 1? Remember that when the randomly generated buying price is higher than the minimal selling price you state here, you will be paid the buying price and the bet is sold. If the bet is sold, you will not see the outcome of the random draw. If the bet is not sold (when the buying price is lower than the selling price), you will partake in the random draw.

The value should be between **0 and 25 and is in pence.**

What is your bet for BOX 2? Remember that when you win your bet you will win 25 pence, but if you lose your bet you will not win anything.

- ○ Red
- ○ Black

What is your minimal reservation price for the bet on BOX 2? Remember that when the randomly generated buying price is higher than the minimal selling price you state here, you will be paid the buying price and the bet is sold. If the bet is sold, you will not see the outcome of the random draw. If the bet is not sold (when the buying price is lower than the selling price), you will partake in the random draw.

The value should be between **0 and 25 and is in pence.**

>>

The random numbers have been drawn and neither of your bets have been sold. However, you won your bet on BOX 1 and you won your bet on BOX 2.

BOX 1:
You have won your bet by betting on Red, which was selected. This means you win 25 pence.

BOX 2: You have won your bet by betting on Red, which was randomly selected. This means you win 25 pence.

As such, **your final reward is 50 pence.**

Outcome:

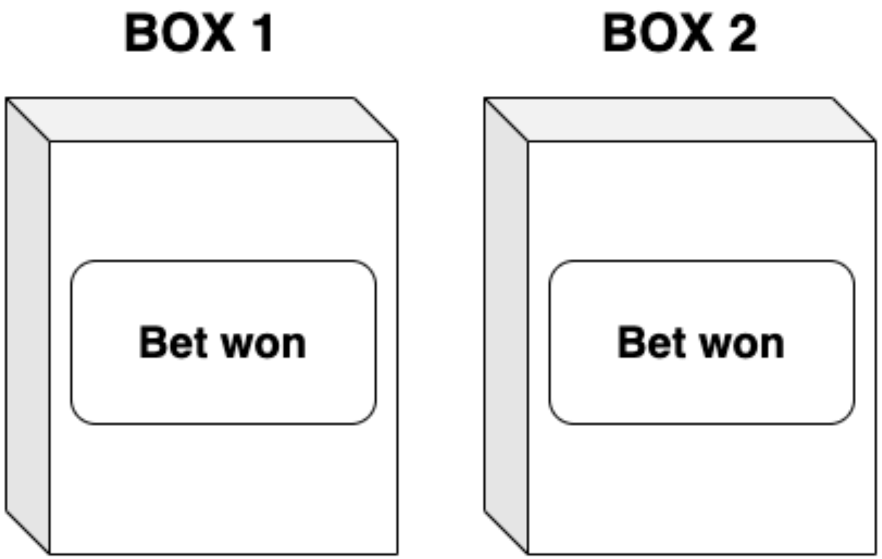

>>

Your final reward for the last two tasks is **50 pence.** Please remember this as you'll be able to make one last choice on the next page: Please write on a piece of paper your total recently won reward.

Remember, you indicated before how much you would donate given certain earnings. Below are your answers from before. You said that, ...

...if you won 14 pence, you said you would donate: 1 pence.
...if you won 28 pence, you said you would donate: 1 pence.
...if you won 42 pence, you said you would donate: 1 pence.
...if you won 56 pence, you said you would donate: 1 pence.
...if you won 70 pence, you said you would donate: 1 pence.
...if you won 84 pence, you said you would donate: 1 pence.
...if you won 98 pence, you said you would donate: 1 pence.

>>

Thank you for completing this last part of the survey. Before finishing this survey, you now have again the option to donate all, some, or none of your earned money to charity below. This charity has again been selected by Giving What We Can, a charity evaluation company, as top charities to donate to in 2021.

However much you end up not donating here will be paid out directly to you after the survey via Prolific.

The charity that you can decide to donate to is the Nuclear Threat Initiative.

Nuclear Threat Initiative
The Nuclear Threat Initiative has as its goal to prevent catastrophic attacks with weapons of mass destruction such as nuclear weapons. It works with governments or non-governmental actors worldwide to prepare for the threat of nuclear war. In their work they aim to strengthen non-proliferation and disarmament agreements and advance international partnerships for nuclear disarmament verification.

According to a survey of experts, the chance of a nuclear war that kills at least 1 million people is about 0.39% every year. It has also been estimated that between 30 and 75 million people would die worldwide as a result of such a nuclear war between the US and Russia. Preparing for such rare events in advance might make them less likely to happen or at least less devastating. The charity's interventions work to reduce this chance by an unknown probability.

Donating to the Nuclear Threat Initiative will help prepare humanity for those very rare but important war risks by reducing the chance of such a conflict, as well as the potential severity of it. This can be achieved by forming new treaties or by fostering international collaboration. Donations are needed every year to have a chance at making an impact in this area.

- ◯ I want to donate.
- ◯ I do not want to donate.

>>

This is the last section of this survey. Please answer below some short questions about yourself and your experience during this survey.

>>

What is your age (in years)?

What is your gender?

- ◯ Male
- ◯ Female
- ◯ Other / Prefer not to say.

What is the highest level of education that you have obtained?

- ◯ High School
- ◯ Undergraduate Degree
- ◯ Graduate/Professional Degree

Are you affiliated with any of the religions below?

If you are affiliated with any religion, do you attend religious service regularly?

- ◯ I am not affiliated with any religion.
- ◯ Yes.
- ◯ No.

Are you married or in a civic partnership?

- ◯ Yes.
- ◯ No.

Do you have children?

- ◯ Yes.
- ◯ No.

How well would you say you yourself are managing financially these days?

- Finding it very difficult.
- Finding it quite difficult.
- Just about getting by.
- Doing alright.
- Living comfortably.

Are you currently employed?

- Full-time
- Part-time
- Unemployed
- Out of the workforce (e.g.retired)

>>

Think about the last time you gave to charity before today. What was most important to you?

- ◯ The total amount given by everyone.
- ◯ The amount that you personally gave.
- ◯ Some other reasons for giving.
- ◯ I have not given to charity before.

>>

Please describe the reasons behind the decisions that you made in the experiment, specifically whether/how much you gave to charity.

Please also describe the reasons behind your decisions in the tasks with the bomb and the two boxes with balls in them.

>>
