# Supplementary material for: Sure-thing vs. probabilistic charitable giving: Experimental evidence on the role of individual differences in risky and ambiguous charitable decision-making
Source: PLoS One. 2022 Sep 22;17(9):e0273971. doi: 10.1371/journal.pone.0273971 (PMC9499298; doi:10.1371/journal.pone.0273971)
Supplement: S2 File — (DOCX) [file pone.0273971.s009.docx]

This document explains variable names from Full Data.

1. First Choice: Charity choice at ‘First Choice’. 0 = No Donation, 1 = SCI Foundation, 2 = GiveDirectly, 3 = Against Malaria Foundation, 4 = Machine Intelligence Research Insitute, 5 = Center for Health Security, 6 = Nuclear Threat Initiative, 7 = context-free Charity 1, 8 = context-free Charity 2 (1-3, 7 = sure-thing charities, 3-6, 8 = probabilistic charities)
2. First Choice Binary: 1 = made a donation, 0 = did not make a donation
3. First Choice STP: 0 = donated to a sure-thing charity, 1 = donated to a probabilistic charity, 2 = did not donate
4. First Donation: amount donated at First Choice in pence
5. Second Choice: Charity choice at ‘Final Choice’. 0 = No Donation, 1 = SCI Foundation, 2 = GiveDirectly, 3 = Against Malaria Foundation, 4 = Machine Intelligence Research Insitute, 5 = Center for Health Security, 6 = Nuclear Threat Initiative (1-3 = sure-thing charities, 3-6 = probabilistic charities)
6. Second Choice STP: 0 = donated to a sure-thing charity, 1 = donated to a probabilistic charity, 2 = did not donate
7. Second Choice Binary: 1 = made a donation, 0 = did not make a donation
8. Second Donation: amount donated at Final Choice in pence
9. BRET_Opened: amount of boxes opened in BRET task
10. BRET_Bomb: 0 = did not select bomb in BRET task, 1 = selected bomb in BRET task
11. Price Risky: Reservation price of risky box in pence
12. Price Ambiguous: Reservation price of ambiguous box in pence
13. Ambiguity Aversion: (Price Ambiguous – Price Risky)*(-1)
14. Impact Sure Thing: Impact judgement of the sure-charity they were shown (from 0-100)
15. Impact Probabilistic: Impact judgement of the probabilistic they were shown (from 0-100)
16. Impact Estimate Sure Thing: Estimation of impact judgements of the sure-charity they were shown (from 0-100)
17. Impact Estimate Probabilistic: Estimation of impact judgements of the probabilistic they were shown (from 0-100)
18. Comprehension Overspend: 0 = participants did not spend more than they earned in Final Choice, 1 = participants did spend more than they earned in Final Choice
19. Attention Check: 0 = Completed attention check, 1 = failed attention check
20. Comprehension Bret: 0 = Completed the comprehension questions, 1 = failed at least one comprehension question
21. Comprehension Boxes: 0 = Completed the comprehension questions, 1 = failed at least one comprehension question
22. Comprehension Binary: 0 = Failed no comprehension/attention questions/checks, 1 = failed at least one comprehension/attention question/check
23. Comprehension Total: Total number of comprehension/attention questions/checks failed
24. Pre 14: Participant statements of future donations if they earned 14 pence after First Choice
25. Pre 28: Participant statements of future donations if they earned 28 pence after First Choice
26. Pre 42: Participant statements of future donations if they earned 42 pence after First Choice
27. Pre 56: Participant statements of future donations if they earned 56 pence after First Choice
28. Pre 70: Participant statements of future donations if they earned 70 pence after First Choice
29. Pre 84: Participant statements of future donations if they earned 84 pence after First Choice
30. Pre 98: Participant statements of future donations if they earned 98 pence after First Choice
31. Numeracy: Total numeracy scores
32. Empathy: Total empathy scores
33. Optimism: Total optimism scores
34. Age: Total age
35. Gender: 0 = Male, 1 = Female, 2 = Other
36. Gender Male: 0 = Not Male, 1 = Male
37. Gender Female: 0 = Not Female, 1 = Female
38. Education: 0 = High School, 1 = Undergraduate Degree, 2 = Postgraduate Degree
39. Education High School: 0 = Not High School, 1 = High School
40. Education Undergraduate: 0 = Not Undergraduate, 1 = Undergraduate
41. Education Postgraduate: 0 = Not Postgraduate, 1 = Postgraduate
42. Religious Affiliation: 0 = No Affiliation, 1 = Protestantism, 2 = Catholicism, 3 = Islam, 4 = Judaism, 5 = Buddhism, 6 = Hinduism, 7 = Sikhism
43. Religion None: 0 = Any Affiliation, 1 = No Affiliation
44. Religion Protestantism: 0 = Not Protestantism, 1 = Protestantism
45. Religion Catholicism: 0 = Not Catholicism, 1 = Catholicism
46. Religion Islam: 0 = Not Islam, 1 = Islam
47. Religion Judaism: 0 = Not Judaism, 1 = Judaism
48. Religion Buddhism: 0 = Not Buddhism, 1 = Buddhism
49. Religion Hinduism: 0 = Not Hinduism, 1 = Hinduism
50. Religion Sikhism: 0 = Not Sikhism, 1 = Sikhism
51. Religious Participation: 0 = No Participation in Religious Activity, 1 = Participation in Religious Activities
52. Marriage Status: 0 = Not Married, 1 = Married
53. Child Status: 0 = Does not have Children, 1 = Has Children
54. Financial Well-being: Likert-scale responses (1-5)
55. Employment: 0 = Unemployed, 1 = Out of Workforce, 1 = Part Time Work, 2 = Full Time Work
56. Employment Unemployed: 0 = Not Unemployed, 1 = Unemployed
57. Employment Out of Workforce: 0 = Not Out of Workforce, 1 = Out of Workforce
58. Employment Part Time: 0 = Not Part Time Work, 1 = Part Time Work
59. Employment Full Time: 0 = Not Full Time Work, 1 = Full Time Work
60. Charity Motivation: 0 = Warm Glow, 1 = Pure Altruism, 3 = Other
61. Motivation Warm Glow: 0 = Not Warm Glow, 1 = Warm Glow
62. Motivation Pure Altruism: 0 = Not Pure Altruism, 1 = Pure Altruism
63. Motivation Other: 0 = Not Other, 1 = Other
64. Random Charity Choice: Random number generated to determine randomisation in experiment. Determines pair of charities shown. 1 = SCI Foundation/Machine Intelligence Research Institute, 2 = SCI Foundation/Center for Health Security, 3 = SCI Foundation/Nuclear Threat Initiative, 4 = GiveDirectly/Machine Intelligence Research Institute, 5 = GiveDirectly/Center for Health Security, 6 = GiveDirectly/Nuclear Threat Initiative, 7 = Against Malaria Foundation/Machine Intelligence Research Institute, 8 = Against Malaria Foundation/Center for Health Security, 9 = Against Malaria Foundation/Nuclear Threat Initiative
65. EV Treatment: Random number generated to determine randomisation in experiment. Determines whether those in the EV Condition are shown the treatment or the control. 1 = Treatment, 2 = Control.
66. Condition: Random number generated to determine randomisation in experiment. Determines condition assignment: 1-7 = Main Condition, 8 = Context-Free Condition, 9-10 = EV Condition
67. Random Charity: Random number generated to determine randomisation in experiment. Determines Charity shown at Final Choice: 1 = SCI Foundation, 2 = GiveDirectly, 3 = Against Malaria Foundation, 4 = Machine Intelligence Research Institute, 5 = Center for Health Security, 6 = Nuclear Threat Initiative
