# Supplementary material for: Sure-thing vs. probabilistic charitable giving: Experimental evidence on the role of individual differences in risky and ambiguous charitable decision-making
Source: PLoS One. 2022 Sep 22;17(9):e0273971. doi: 10.1371/journal.pone.0273971 (PMC9499298; doi:10.1371/journal.pone.0273971)
Supplement: Attachment — Submitted filename: Response to Reviewers.docx [file pone.0273971.s012.docx]

**Reviewer #1**

**Summary Comment:**

In this article, the authors examined the effect of several individual difference measures, among them, risk and ambiguity preferences, numeracy, empathy, optimism, and donation motivation. The main dependent variable is if and if then how much participants were willing to donate from their experimental endowment to one of two different types of charities, which the authors call sure-thing or probabilistic charities. Another dependent variable was the donation to an individual charity (either sure-thing or probabilistic) at a later stage of the experiment. In addition, the authors examined whether an intervention, namely advertising the EV-maximizing principle as a decision criterion, has an effect on donation choices and whether presenting anonymous and context-free donations rather than real-world charities with concrete description of their cause lead to different behavior than the main condition. As results, none of the individual difference measures significantly affected the choice of donating to a sure-thing or probabilistic charity, and only empathy and donation type affected overall donation in the choice to donate to an individual charity in some models.

The paper is clearly written and reports all methods, hypotheses and analyses in an understandable fashion. I appreciate that the study was pre-registered and I think the general research question is important and interesting. Moreover, the individual difference measures are all taken from previous research and seemed to be reasonable choices to measure the respective constructs. However, I am not fully convinced of the classification into sure-thing and probabilistic charity, of the theoretical underpinning of the hypotheses, and the power of the study. I will discuss each of these issues in more detail in the next section.

**Comment 1:**

The main dependent variable is the choice between so-called sure-thing and probabilistic charities. In total there were six charities, three classified as sure-thing and three classified as probabilistic. This classification is rather ad-hoc and neither rooted in empirical evidence, nor based on principled theoretical arguments. Whereas I might agree from reading the description that this classification could be justified, **the current study did not establish that all or even the majority of participants perceived the charities as assumed in the classification.** Instead, there is a lot of information in the descriptions of the charities and participants might focus on different information than impact probability. Second, participants could perceive other information about the charity as varying in riskiness. For example, the direct money transfer could be perceived as risky with respect to the ultimate use of the donated money. Finally, the charities in the different conditions could also differ on other dimensions than riskiness. For example, the sure-outcome charities all have a direct impact on individual people, whereas the probabilistic charities have a more indirect impact on a large amount of people. To be fair, the authors mention concerns about the classification of charities into sure-thing and probabilistic in the discussion. However, the provided evidence in favor of their classification are not convincing as they do not directly speak towards a difference in perceived riskiness between the two types of charity. **Instead, a straight-forward way to examine this question empirically would be to ask participants either from the old participant pool if available or a new one, how much they rate the respective charities on several dimensions including the riskiness of the project.**

**Response 1:** Thank you very much for raising this critique and for suggesting such an actionable step. As a first step, we have improved on our theoretical justification of this distinction: We have elaborated in much more detail than before for why one might think theoretically that these charities can be divided into these two groups. Furthermore, following your recommendation we have now conducted an auxiliary study (n=101) where participants rated the charities based on how risky their interventions were (riskiness) and how quantifiable they thought these interventions were (ambiguity). We found that probabilistic charities were rated as significantly more risky than sure-thing charities (p<.001) and that they were also rated as significantly more difficult to quantify (i.e. as more ambiguous) (p<.001), indicating that participants do in fact pick up on the underlying dimension of variation in probability and ambiguity in the predicted direction. Taken together, we believe that this distinction is now much better justified than in the original manuscript both theoretically and empirically. This, we argue, allows for a relatively straight interpretation of the results and we hope that this allays most of your concerns about this distinction. However, we will remain to call attention to this potential issue in our discussion section to ensure that all readers are made aware of the potential design weakness and the evidence presented here such that they can form an adequate picture of the data presented in this paper. However, recall also that moving the level of riskiness from easily controllable aspects like donation in standard lab settings to the actual charities themselves is one of the contributions of this paper.

**Example (p. 34-35):**

**Appendix A – Auxiliary Study**

We conducted a post-hoc auxiliary study to empirically confirm our categorisation into sure-thing and probabilistic charities. This is to ensure that this distinction is not only theoretically grounded but also perceived as intended by the general public. We recruited a total of 101 participants on Prolific that had not participated in the main study, none of which failed the attention check. Participants were paid £0.75 for their participation. They were presented with all six charities and were asked to rate them on a scale from 0-10 on the likelihood that the charity’s intervention succeeds (relating to uncertainty over its interventions) and on the quantifiability of the charity’s intervention (relating to ambiguity). We also asked participants to rate the charities on their moral deservingness to keep the objective of this study opaque.

We find strong support for the distinction between sure-thing charities and probabilistic charities on the basis of both uncertainty and ambiguity. See Appendix Table 1 for means, standard deviations, and medians of the uncertainty and ambiguity ratings for all six charities, with 0 indicating low probability that the charity’s intervention will succeed and a low level of quantifiability of its interventions, and 10 indicating a high probability and quantifiability. In other words, the higher the scores, the less risky and the less ambiguous the charity’s respective intervention is.

Appendix Table 1—Probability-Ratings for all Six Charities

| Uncertainty |  |  |
| --- | --- | --- |
|  | Mean (SD) | Median |
| Sure-Thing Charity 1 (SCI Foundation) | 7.19 (2.08) | 8 |
|  |  |  |
| Sure-Thing Charity 2 (GiveDirectly) | 5.92 (2.09) | 6 |
|  |  |  |
| Sure-Thing Charity 3 (Against Malaria Foundation) | 7.36 (1.83) | 8 |
|  |  |  |
| Probabilistic Charity 1 (Machine Intelligence Research Institute) | 4.15 (2.46) | 4 |
|  |  |  |
| Probabilistic Charity 2 (Nuclear Threat Initiative)  fd | 4.09 (2.64) | 4 |
|  |  |  |
| Probabilistic Charity 3 (The Center for Health Security) | 4.30 (2.50) | 4 |
|  |  |  |
|  |  |  |
| Ambiguity |  |  |
|  | Mean (SD) | Median |
|  |  |  |
| Sure-Thing Charity 1 (SCI Foundation) | 7.28 (1.97) | 8 |
|  |  |  |
| Sure-Thing Charity 2 (GiveDirectly) | 5.42 (2.49) | 5 |
|  |  |  |
| Sure-Thing Charity 3 (Against Malaria Foundation) | 7.24 (2.10) | 8 |
|  |  |  |
| Probabilistic Charity 1 (Machine Intelligence Research Institute) | 3.63 (2.72) | 3 |
|  |  |  |
| Probabilistic Charity 2 (Nuclear Threat Initiative)  fd | 4.03 (2.73) | 4 |
|  |  |  |
| Probabilistic Charity 3 (The Center for Health Security) | 4.69 (2.40) | 4 |
|  |  |  |

*Notes:* Mean, Standard Deviation, and Median of risk and ambiguity ratings for all six charities.

We find that the data behave as generally expected, with sure-thing charities receiving higher ratings about the likelihood that their interventions will succeed as well as higher ratings for the quantifiability of their interventions, and probabilistic charities receiving lower rating correspondingly. Adding a subject’s scores for the individual charities of each bucket, we find that sure-thing charities are rated as having significantly higher probability interventions (M=20.47, SD=4.52) than the probabilistic charities (M=13.96, SD=5.83). This difference, 6.51, 95% CI [5.19, 7.82] was highly statistically significant, t(100)=9.81, p<.001. The same picture emerges with regard to the quantifiability of the interventions, with the mean of the sum of the quantifiability scores of sure-thing charities (M=19.83, SD=4.60) being significantly higher than that of the probabilistic charities (M=12.85, SD=6.70), with the difference of 6.98, 95% CI [5.62, 8.34] also being statistically significant at t(100)=10.17, p<.001. The effect sizes of these two differences, in Cohen’s d, is d=.98 for the probability ratings and d=1.01 for the quantifiability rankings. This provides strong support for our theoretically based distinction between sure-thing and probabilistic charities.

**Comment 2:**

Since there were different charities administered in the stimulus material, it would make sense to **estimate regressions with stimulus random effects, instead of OLS**. Other models that cluster errors on the stimulus level might also be possible. **Similar to the logistic, this could be done as a robustness test.**

**Response 2:** Thank you very much for suggesting this analysis. We agree that this type of regression would be appropriate for our data structure. We now report one such model in the appendix (Appendix Table 3, Model 5), where we find the same pattern of results as in the main analyses, suggesting that our results are also robust to this modelling choice.

**Example (p. 36-37):**

We also report an additional robustness check of Model (1) in Appendix Table 3, Model (5). Specifically, we report a random effects model with the stimulus material being treated as a random effect. As we had three sure-thing and three probabilistic charities, there were nine charity pairs that participants could have been presented with. In Model (5), we treated the stimulus (i.e. the charity pair presented) as a random effect (with nine levels). Because significance levels are not unproblematic in mixed models like this, we also report 95% confidence intervals of the estimates. The results indicate that our null effect is robust to this model choice as well.

Appendix Table 3— Regression Results For Main Choice – Random Effects Robustness Check
Predicting Choice between Sure-Thing and Probabilistic Charities

|  | (5) |  |
| --- | --- | --- |
| Risk Attitude | .002 (.005) | [-.008, .012] |
|  |  |  |
| Ambiguity Aversion | -.002 (.005) | [-.010, .006) |
|  |  |  |
| Numeracy | .003 (.023) | [-.042, 0.47] |
|  |  |  |
| Empathy | .001 (.002) | [-.005, .004] |
|  |  |  |
| Optimism | -.006* (.003) | [-.012, .001] |
|  |  |  |
| Donor Type |  |  |
| Warm-Glow | -.007 (.047) | [-.099, .086] |
| Pure Altruism | -.040 (.059) | [-.156, .077] |
|  |  |  |
| Age | .001 (.002) | [-.003, .004] |
|  |  |  |
| Gender | -.024 (.047) | [-.115, .068] |
|  |  |  |
| Education |  |  |
| Undergraduate degree | .041 (.048) | [-.053, .134] |
| Postgraduate/Professional degree | -.023 (.051) | [-.123, .078] |
|  |  |  |
| Religion |  |  |
| Protestantism | -.043 (.062) | [-.165, .080] |
| Catholicism | .020 (.068) | [-.114, .154] |
| Islam | -.035 (.113) | [-.246, .188] |
| Judaism | -.006 (.354) | [-.691, .703] |
| Buddhism | -.060 (.265) | [-.581, .462] |
| Hinduism | .325 (.254) | [-.175, .825] |
|  |  |  |
| Religious Participation | -.004 (.083) | [-.168, .159] |
|  |  |  |
| Marriage Status | -.019 (.046) | [-.071, .108] |
|  |  |  |
| Children | .009 (.050) | [-.090, .107] |
|  |  |  |
| Financial Wellbeing | .009 (.022) | [-.035, .053] |
|  |  |  |
| Employment |  |  |
| Part-time employment | .058 (.054) | [-.047, .164] |
| Full-time employment | .015 (.049) | [-.083, .112] |
|  |  |  |
| Sample size | 307 |  |

*Notes:* Random effects model, coefficients, and standard errors, as well as 95% CIs. *p<.1, **p<.05, ***p<.01, ****p<.001

**Comment 3:**

**More effort should be spent on the theory of why the examined individual differences should be correlated with the choice between the sure-thing and the probabilistic charity**. In particular, why should risk preference be related to this choice, if—as mentioned by the authors—previous literature (Vives & FeldmanHall, 2018) did not find a connection between risk-preference and prosocial behavior, that the exact probabilities of success of a charity are most likely ambiguous and that risk- and ambiguity preferences are not strongly correlated? Similarly, the connection between optimism or numeracy and the main DV is not build on theoretical considerations nor on previous findings that suggest such a connection. **A strong foundation of the hypotheses would considerably strengthen the importance of the article.**

**Response 3:** Thank you very much for pressing us on this. We agree that our explanations were not thorough enough in the previous manuscript. We have now expanded upon our justification for all our individual difference measures throughout the revised manuscript, making clearer our reasoning for their inclusion. Specifically, we have now backed up the inclusion of all items with a wider number of references to the literature, showing how all constructs have been previously found to be associated with some measure of pro-social behaviour or charitable giving to directly motivate and justify their inclusion. Further, we have also updated our analytical reasoning to further improve the justifications of inclusion on both counts.

**Example (p. 6-7):**

First, we investigate whether donor choices can be explained by individual differences in risk and ambiguity attitudes. Previous work in domains such as stock market participation (Bianchi & Tallon, 2018) and health-related field behaviours (Sutter et al., 2013) has found that attitudes to risk and ambiguity can play significant roles. In context of pro-social behaviour in game environments the results show that ambiguity aversion may play a role (while risk aversion sometimes does not) (Vives & FeldmanHall, 2018), though risk aversion has also been found to be “predictive for giving” (Cettolin, Riedl, & Tran, 2017, 95). As such, we argue that given risk and ambiguity aversion have been shown to impact behaviour in many contexts including charitable giving, this makes it an a priori interesting relation to test. This hypothesis is also theoretically grounded, in that it might be the case that one’s preference not to give to charities that have a low chance of making an impact might be driven by an individual’s general risk aversion profile, or it might be that given the ambiguous nature of charitable interventions that it is only ambiguity aversion that impacts this choice.

[…]

For example, previous research has found that those lower in numeracy were more insensitive to proportions of donation recipients (Kleber, Dickert, Peters, & Florack, 2013) and that they showed higher susceptibility to changes in numeric presentation (Dickert, Kleber, Peters, & Slovic, 2011). It may as such be the case that one’s level of numeracy also meaningfully impacts behaviour in the context studied here as the probabilistic charities include interventions that have a small chance of making a large impact. Understanding these proportions plausibly requires a certain level of numeracy. Further, one may also think that a general proclivity to optimism may bias individuals towards overestimating the success of probabilistic charitable interventions, or conversely that higher pessimism may explain a preference for sure-thing charities as those promise to have a reliable impact even in the worst-case scenario. This is corroborated by previous research that draws on the German socioeconomic panel and finds that optimism predicts charitable giving in some of their models (Boenigk & Mayr, 2016).

**Comment 4:**

There is some uncertainty about the power of the regression results as the pre-registered power analyses were based on the assumption that 2/3 donated, but the true number of donations was only 1/3. **I appreciate that this is mentioned in the discussion, but I think this aspect deserves a bit more attention**. In particular the EV-max intervention and the context-free manipulations with a much smaller sample size than the main dependent variable, might not have enough power to draw definite conclusions. **Instead, it might make sense to put these analyses in an appendix and label these analyses as inconclusive.**

**Response 4:** Thank you for your comment and the actionable advice. We agree with you that the context-free manipulations (and in some respects the EV-information interventions) might have a too-low sample size to draw conclusions from justifiable. We have now followed your suggestion and labelled these as ‘inconclusive’ and have put them in the appendix. However, we do not share the same pessimism about Main Choice: Recall that our original power analysis was extremely strict, having been aimed to have .95 power to detect at f2=.02. While we understand the statistical challenges of calculating power post-hoc, moving from .95 power to .8 in the above power analysis would have reduced the sample size needed to nearly a fifth, making our collected sample size much more reasonable with this in mind. Also, our data from Final Choice does not share these concerns, and following Reviewer 2, we have now increased the prominence of our discussion of the results from Final Choice.

**Example (p. 17):**

However, because the number of people who made donations was unexpectedly small, both of these conditions did not have the power that we calculated prior to running this study to detect a meaningful effect. This means that results of these conditions are inconclusive. We still report the full pre-registered analyses in the appendix, see Appendix D, but do not discuss them in the main results and discussion sections.

**Comment 5:** For the main DV, I appreciate that the authors calculate equivalent tests to examine the measurement uncertainty around the true effect. **In my view, a better or at least complementary approach would be to calculate Bayes factors to evaluate the evidence for the Null (see Jarosz & Wiley, 2014; Rouder & Morey, 2012).** Bayes Factors could clearly state whether enough evidence has been collected to conclude that there is no effect of an individual difference measure on the choice between the sure-thing or the probabilistic charity. In case that the evidence is inconclusive, it might make sense to collect more data.

**Response 5:** We are very glad that you appreciate our use of equivalence tests. We have now expanded our usage of equivalence tests (now also for the results from Final Choice) to provide the reader with a wide range of plausible test ranges that may allow us to conclude a null effect (and to narrowly estimate the range of the equivalence bounds). Further, we have also followed your suggestion and included Bayesian analyses following Rouder & Morey (2012). The Bayesian results corroborate our equivalence test results in that they provide strong evidence in favour of a null effect.

**Example (p. 24):**

Following Rouder & Morey (2012), we compute exploratory Bayes factors to evaluate the evidence for the null. We report Bayes factors for linear models based on Liang et al. (2008), using a Jeffrey-Zeelner-Siow mixture of g-priors. Applying the default r scale of .353 for the results regarding Main Choice in Model (1), we calculate a JZS Bayes Factor of 2.49*10^7^, suggesting very strong evidence in favour of the null hypothesis. The same analysis regarding Final Choice in Model (2) also provides evidence in favour of the null, though at a significantly smaller magnitude, at a JZS Bayes Factor of 22.15. This corroborates the results of the equivalence tests and suggests that our data provide strong evidence in favour of a robust null result.

**Comment 6:**

**I would suggest to describe the hypotheses in the introduction in terms of the alternative hypothesis.** Presenting hypotheses as the Null makes the text wordier and more complex than necessary.

**Response 6:** Thank you for your comment! While we agree that statements in terms of alternative hypotheses would aid comprehension in some respects, we will retain the phrasing in terms of null hypotheses for the following reasons:

1) We have pre-registered the null hypotheses this way and would like to stick as closely as possible to our pre-registration.
2) More importantly, because the results in this paper are primarily null results, retaining the null hypothesis structure enables us to more meaningfully discuss our inability to reject the null and to discuss evidence in favour of the null (with equivalence tests). As such, while alternative hypotheses might be easier to understand in the earlier sections of the paper, we believe that when it comes to the statistical results and discussions of them, null hypothesis phrasings are actually more straightforward.

**Comment 7:**

On page 5 it is stated that:

“Our research builds on this literature but is importantly different, primarily because of our focus on donations to actual charities and not on pro-social behaviour in abstract games.”
And on page 6:

“Specifically, we focus on donation behaviour between real world charities that are made with an earned endowment, in contrast to abstract laboratory game pro-social decisions and hypothetical choices.”

These comments suggest that there is basically no research about charitable giving and all research about social preferences is only done in abstract experimental paradigms. **I think this is a wrong impression and literature about charitable giving should be properly cited in these situations**. Possible literature and review articles of which some are also cited in the text are Bekkers and Wiepking (2011), Karlan and List (2007), and Vesterlund and Sonnevi (2007).

References
Bekkers, R., & Wiepking, P. (2011). A literature review of empirical studies of philanthropy: Eight mechanisms that drive charitable giving. Nonprofit and voluntary sector quarterly, 40(5), 924-973.

Jarosz, A. F., & Wiley, J. (2014). What are the odds? A practical guide to computing and reporting Bayes factors. The Journal of Problem Solving, 7(1), 2.

Karlan, D., & List, J. A. (2007). Does price matter in charitable giving? Evidence from a large-scale natural field experiment. American Economic Review, 97(5), 1774-1793.

Rouder, J. N., & Morey, R. D. (2012). Default Bayes factors for model selection in regression. Multivariate Behavioral Research, 47(6), 877-903.

Vesterlund, L., & Sonnevi, G. (2006). 24. Why Do People Give?. In The nonprofit sector (pp. 568-588). Yale University Press.

Vives, M. L., & FeldmanHall, O. (2018). Tolerance to ambiguous uncertainty predicts prosocial behavior. Nature communications, 9(1), 1-9.

**Response 7:** Thank you very much for pressing us on this! We of course did not mean to suggest that there was no research fitting these criteria. We have now adjusted our language throughout the literature review and cited all the literature outlined by you (except the ones that we had already cited before), also clearing up some additional issues in these sections: Specifically, we now make explicit that our contribution is primarily moving risk over donations in tightly controlled abstract games (as has been done before) to the risk/ambiguity over actual charities’ interventions, something that has both not been studied in detail and is much closer to the actual choice environment that potential donors find themselves in. Now we make clear that our claim to increase ecological validity is with regards to this aspect specifically, and of course not to charitable giving overall (though we agree that our previous manuscript did not make this sufficiently clear). We hope that our updated language now removes all ambiguity regarding this issue.

**Example (p. 4-5):**

Our research builds on the literature on risk that has so far mostly employed directly controllable levels of risk in the lab. For example, in abstract game scenarios, risk can be controlled and stated precisely, for example by imposing a 50% chance of one’s donation not going through, or by introducing a 5% chance that one’s donation is matched. Crucially though, our research is substantially different from the discussed literature primarily because we move the level of risk from directly calculable interventions in the lab (as outlined above) to the actual charities themselves. While this introduces several design challenges, we argue that this step leads to an increased level of ecological validity of any potential finding. However, note that there is already a large literature on charitable giving generally that has a similar or higher level of external validity (Bekkers & Wiepking, 2011; Karlan & List, 2007; Vesterlund & Sonevi, 2006). However, our paper’s main contribution is the moving of our focus on risk and ambiguity to actual organisations and their interventions and away from aspects that can be controlled in the lab. Having risk and ambiguity at the level of actual charities is the level at which risk and ambiguity typically enter people’s decision-making processes; rarely are we uncertain as to whether our donation will randomly increase when we donate (as in some experimental lab studies), but we are almost always acting under uncertainty about the charity’s interventions that we consider donating to.

**Reviewer #2:**

**Summary Comment:**

While I was reading the paper, I found the research questions interesting and important. I liked it a lot. However, when I read though the experiment design, I found, unfortunately, the design in the Main choice and context-free choice is not appropriate to answer the research questions. The concern is that the current design asks the participants to make two choices: which type of charity to donate to and how much to donate, while these two decisions are correlated with each other. Consequently, the experiment is not well controlled.

**Comment 1:**

To make this more clear, I’ll first describe what is an ideal design and then point out the issues about the current design. To answer the research questions or test the hypotheses, we can either test whether they prefer to donate to sure-thing charity or probabilistic charity or test how much they would like to donate given sure-thing charity or probabilistic charity. In the former case, we need to control for the donation level, i.e. if they donate, they donate the same amount of money. In the latter case, we randomly control for the charity type. The latter case is of course the Final Choice design in the current paper which I fully agree is the correct way.

The issue with the current design is that, theoretically, there should be a point where one is indifferent between donate a certain amount of money under sure-thing charity and another amount under probabilistic charity. So what we watched in current experiment is just one of the two choices they are indifferent from. Therefore, it is hard to tell from their preference for the charity type.

To extend the above point a bit more and perhaps it helps to make it more clear, suppose there are some amount of people who would donate zero no matter what type of charity they are assigned or chose in the Main choice (this happens I guess for sure because there are only 35.8% of participants made a donation). For these people, economically, their choice of the charity type is invalid as the cost is zero (they will not donate anyway). This is an extreme case.

Now return to the paper**, to save it, first, I think the Final Choice design is good, the authors may want to rely more on the data generate from the Final Choice.** But at the same time take it in mind that this is last task which may suffer from order or experimenter demand effect.

**Response 1:** Thank you very much for your thoughtful comments. We actually agree with you that the design for Final Choice is superior to that of Main Choice, primarily because Main Choice is a more substantive (and thus more confoundable) design, compared to the overall much cleaner design of Final Choice. As such, we have followed your recommendation and have prioritised the data from Final Choice. Specifically, we now report Main Choice and Final Choice results next to each other throughout the entire manuscript and discuss their comparative strengths and weaknesses in the discussion section. Further, on suggestion of Reviewer 1, we have also moved the results and discussion of the no-context condition as well as the expected-value treatment to the appendix, giving the Final Choice data even more space (effectively doubling its share of discussion space in the main manuscript). However, we will not solely rely on the data from Final Choice as we pre-registered our main hypotheses with both Main Choice and Final Choice in mind, and because we do believe that the data from Main Choice do meaningfully contribute to our understanding of the research question. We hope that our restructuring and comparative focus on Final Choice satisfactorily responds to your actionable request to rely more on Final Choice.

Lastly, we believe that given we have understood your worry correctly, we have some empirical evidence from our data that should go towards alleviating your worry to some extent. We believe that one testable prediction from your worry would be that those who donated to sure-thing charities before (i.e., in Main Choice) should donate less to probabilistic charities at Final Choice. We have investigated this in our data, looking at participants who donated to a sure-thing charity in Main Choice and correlated these donations with their donation amounts (including 0) when they were presented with a probabilistic charity. We do not find a statistically significant correlation at r(184)=.087, p=.238. We also do not find the predicted negative correlation in the reverse (with participants who donated to a probabilistic charity first and were then shown a sure-thing charity in Final Choice, though these results rest on a very low sample size and should probably be disregarded), with r(25)=.442, p=.027. These results suggest that your worry may not impact the data as much as one might have thought, making our solution of presenting both Main Choice and Final Choice as central pieces of the paper justified. Further, when you claim that one piece of your worry is that some people would “donate zero no matter what type of charity they are assigned or chose in the Main choice” we just want to quickly point out that those people would not be included in any of the analyses of Main Choice as only those making a donation are included in the regression models based on our pre-registered exclusion criteria.

**Example (p. 6; 24-25):**

In our experiment, each participant is first presented with a randomly selected pair of charities consisting of one charity of each type to control for accidental confounds relating the charity’s context as each are presented with substantial additional accurate information to increase the naturalness of the choice. Participant choices with respect to this randomly selected charity pair then allows us to isolate and capture the element of probability between the two charity types. In the second part of this experiment, we study participant behaviour when they are shown only one randomly selected charity (either sure-thing or probabilistic), which more narrowly captures the predictive value of individual differences on donation choices to charities of specific types. Overall, we find little to no evidence that individual differences in risk/ambiguity attitudes, numeracy, optimism, and donor type predict charitable giving behaviour. However, we do find that a purely altruistic donor type predicts donations to probabilistic charities. As such, we take this paper to be primarily reporting a null-result.

[…]

Overall, we find little or no evidence in favour of rejecting our null hypotheses, and as such this paper primarily reports a negative result: First, we do not find evidence that risk and ambiguity attitudes robustly predict charitable decision-making between sure-thing and probabilistic charities (null hypothesis #1), and we also do not find evidence that individual differences in numeracy, optimism, donor type, and empathy predict behaviour in this choice as well (null hypothesis #2). Further, we only find weak evidence predicting behaviour when participants are presented with either a sure-thing or a probabilistic charity, where we find that pure altruistic donor types significantly predict donation frequency to probabilistic charities (null hypothesis #5). Further, due to a lower than planned sample size, we are unable to conclusively evaluate the data about our no-context condition (null hypothesis #3) and with regard to the effect of the informational treatment introduced participants to expected-value reasoning (null hypothesis #4).

[…]

As such, it may be justified to put more interpretative emphasis on the results from Final Choice compared to Main Choice.

**Comment 2:**

**Second, if the authors really want to use the data from the Main Choice, they should at least control for the donate amount in the regression.** Though I still think this is not valid enough to test the hypotheses.

**Response 2:** Thank you for your comment. However, we have decided not to follow it because we are mindful of introducing endogeneity in the regression models by having two dimensions of the same choice in the same model as independent and dependent variables (choice of charity as Dep. V and size of that donation choice as Ind. V). If there is an opportunity for further revision, could you please elaborate for why you think we should include this control variable despite the challenges of endogeneity (and how we could circumvent these)? Then we’d be more than happy to follow your advice.

**Comment 3:**

In table 4, to test the hypothesis using the Final Choice data, **they should add in interactive term between treatment variable (sure-thing or probabilistic) and the major explanatory variables** to test the difference in difference, that is whether the major explanatory variables can explain the donation difference under sure-thing or probabilistic charity (the major question this paper intends to answer).

**Response 3:** Thank you very much for this comment! We agree that this would be a better approach and have moved our original (pre-registered) regressions to the appendix and now report two regressions with the interaction terms as proposed by you in the main text and rely on their results throughout the paper. We also use the interaction terms for our equivalence tests of the Final Choice coefficients.

**Example (p. 21-22):**

Second, we investigate general donation behaviour in Final Choice where participants were either presented with a sure-thing or a probabilistic charity. Here, we report two further regression models relevant to null hypothesis #5. These are not the pre-registered ones but instead include interaction terms that we did not pre-register. For specifications of the regression models as pre-registered, see Appendix C (Appendix Table 6). Model (2) explains frequency of donation, and Model (3) predicts size of donation. For Model (2), not making a donation is coded as 0 and making a donation is coded as 1. The central variables are interaction terms, where we interact the main individual difference measures with the condition. Specifically, they are coded with 0 = sure-thing charity and 1 = probabilistic charity for all our main explanatory variables. In these analyses, participants from all conditions are included and are split only by which type of charity they were presented with at Final Choice; recall that each participant here was only presented with one randomly selected charity.

Table 3—Regression Results for Charitable Giving Behavior in Final Choice
Predicting Frequency of Giving and Size of Donation

|  | (2) | (3) |
| --- | --- | --- |
| Risk Attitude | .002 (.005) | .124 (.120) |
|  |  |  |
| Ambiguity Aversion | -.002 (.004) | -.127 (.109) |
|  |  |  |
| Numeracy | .015 (.017) | -.193 (.453) |
|  |  |  |
| Empathy | .007**** (.002) | .212**** (.048) |
|  |  |  |
| Optimism | .003 (.003) | .062 (.071) |
|  |  |  |
| Donor Type |  |  |
| Warm-Glow | .061* (.047) | 2.170* (1.237) |
| Pure Altruism | .045 (.058) | -.144 (1.525) |
|  |  |  |
| Condition X Risk Attitude | .003 (.006) | .019 (.161) |
|  |  |  |
| Condition X Ambiguity Aversion | .002 (.005) | .085 (.143) |
|  |  |  |
| Condition X Numeracy | .006 (.018) | .275 (.482) |
|  |  |  |
| Condition X Empathy | -.002* (.002) | -.086 (.056) |
|  |  |  |
| Condition X Optimism | -.004 (.003) | -.033 (.088) |
|  |  |  |
| Donor Type |  |  |
| Condition X Warm-Glow | -.034 (.063) | -.993 (1.649) |
| Condition X Pure Altruism | .177** (.083) | 2.955 (2.176) |
|  |  |  |
| Age | .001 (.001) | .047 (.030) |
|  |  |  |
| Gender | -.050* (.030) | -.747 (.788) |
|  |  |  |
| Education |  |  |
| Undergraduate degree | -.050 (.031) | -1.129 (.800) |
| Postgraduate/Professional degree | .030 (.034) | -.095 (.897) |
|  |  |  |
| Religion |  |  |
| Protestantism | .071* (.043) | .153 (1.129) |
| Catholicism | .045 (.047) | 1.468 (1.239) |
| Islam | .173* (.142) | 6.606*** (2.317) |
| Judaism | .134 (.142) | 2.636 (3.730) |
| Buddhism | .159 (.160) | 4.994 (4.207) |
| Hinduism | .149 (.126) | 7.049** (3.301) |
| Sikhism | .046 (.260) | -2.343 (6.819) |
|  |  |  |
| Religious Participation | .015 (.054) | -.265 (1.406) |
|  |  |  |
| Marriage Status | -.025 (.031) | -.469 (.819) |
|  |  |  |
| Children | .032 (.033) | .697 (.877) |
|  |  |  |
| Financial Wellbeing | .023 (.015) | .398 (.384) |
|  |  |  |
| Employment |  |  |
| Out of the workforce | .017 (.055) | -.820 (1.442) |
| Part-time employment | -.019 (.047) | -2.211* (1.243) |
| Full-time employment | -.029 (.042) | -1.436 (1.107) |
|  |  |  |
| R^2^ | .076 | .085 |
|  |  |  |
| Sample size | 1177 | 1177 |

*Notes:* OLS regressions reporting unstandardised coefficients and standard errors. Model (2) predicts frequency of donation and Model (3) predicts size of donation. Interaction terms interact the condition (0 = sure-thing charity, 1 = probabilistic charity) with the explanatory variables *p<.1, **p<.05, ***p<.01, ****p<.001

Here we find that while empathy predicts donation frequency and size overall, it is only the interaction term with pure altruism that statistically significantly predicts frequency of donation to probabilistic charities. This is in line with theoretical predictions that hold that pure altruists would be more likely to give to probabilistic charities as they primarily care about the impact on social welfare. Further, in our robustness check that reproduces Model (2) as a logit model (Appendix C, Appendix Table 5), we find the same result. All other pre-registered variables (interacting with condition) do not predict donor behaviour.

**Comment 4:**

**The paper, especially the abstract can be shortened to make it more readable.**

**Response 4:** Thank you for your comment. In this revision we have tried to make the paper shorter throughout (and focused especially on the abstract). Most of the robustness checks that were requested by the reviewers are now in the appendix to ensure that the paper’s length is not massively increased by responding to reviewer comments. The paper without references and appendix is now under 30 pages.

**Comment 5:**

**It is helpful to report R2 in the regression** to show how much can be explained by the factors measured in this study.

**Response 5:** Thank you very much for suggesting this. We now report R^2^ (or Cox and Snell R^2^) for all our regressions.

**Comment 6:**

**Typos**: by conducting an a priori power analysis

**Response 6:** Apologies, but we are unsure what the typo here is.
