# Supplementary material for: Sure-thing vs. probabilistic charitable giving: Experimental evidence on the role of individual differences in risky and ambiguous charitable decision-making
Source: PLoS One. 2022 Sep 22;17(9):e0273971. doi: 10.1371/journal.pone.0273971 (PMC9499298; doi:10.1371/journal.pone.0273971)
Supplement: Attachment — Submitted filename: Respopnse to Reviewers.docx [file pone.0273971.s013.docx]

Dear Reviewers,

Thank you very much for evaluating our revised manuscript and for giving us the opportunity to revise and improve it further.

We are very happy that both of you agreed that our previous revision “improved the paper considerably” (R1) and “improved the paper a lot” (R2). However, we acknowledge that we have not yet fully addressed all of the comments raised to a satisfactory extent and we hope that in this revision, we will have done so.

Overall, we have made the following general improvements.

Following Reviewer 1’s recommendations, we have now moved our auxiliary study into the main text to give it more spotlight compared to the previous appendix placement. We have also updated our Bayesian analyses, now reporting comparisons of all individual models with the intercept-only model. Further, in part in response to Reviewer 2’s comment from the first round of revision, we have also provided model-averaged coefficients for the full Bayesian models of Final Choice to provide further analyses of the condition that suffers less design worries.

Following Reviewer 2’s recommendations, we now control for the amount donated in all analyses of Main Choice (and have updated our equivalence tests accordingly) such that Main Choice’s design downsides are at least in part addressed. In the discussion section, we now also discuss in much detail the major limitation that Reviewer 2 has identified with Main Choice. While we cannot drop these analyses from our paper as they were our primary pre-registered analyses, we now give significant space to this objection such that readers are fully informed about this design choice and its flaws. Further, we now report a regression in the appendix that mirrors Main Choice but aims to control for some of those worries.

Additionally, we have slightly reworked the literature section, updated the parts of the discussion section, and made further minor changes throughout the document.

We hope that these changes are sufficient for this paper to be considered for publication at PLOS One. However, if you have any further improvements that you’d like to see us make, we’d be more than happy to accommodate them too!

**Reviewer #1**

**General Comment:** In general, the authors incorporated all my comments and improved the paper considerably. I would like to thank and congratulate them for their work and their constructive responses to the raised comments. I think the paper can be published with only minor revisions:

**General Response:** Thank you very much! We hope that the changes below properly respond to your requested revisions.

**Comment 1:** I very much like the additional study and the demonstration that participants indeed perceived the one class of charities as riskier and more ambiguous than the other. Given the importance of this result in judging the whole validity of the study with respect to the research question, I feel this study deserves much more space in the main manuscript. I think it should get a small methods and results section (maybe called study to validate the stimuli) and should also be mentioned already in the Methods of the main study when the different charities are introduced as stimuli. Importantly, while the additional study helps to support the claim that the charities differ in perceived riskiness, it does not exclude the possibility that the groups of charities also differed on other dimensions (e.g., individual vs. group as recipients etc.). I think this is an important limitation that should be mentioned in the discussion.

**Response 1:** Glad to hear the addition of the auxiliary study has adequately responded to your previous request for revisions. We have now moved the study to the main text as you suggested. We have also mentioned the valid concern you have raised in the discussion section.

**Example (p. 30):** However, it is worth pointing out that this auxiliary study cannot help us rule out whether the two groups of charities also differed along other dimensions in the perceptions of participants.

**Comment 2:** It is great that the authors conducted a Bayes Factor analysis. However, it was not exactly clear to me which models the authors compared. If it were the full model with all predictors vs. the null model with only an intercept this should be clearly reported and interpreted accordingly. Given the hypotheses, it would make sense to compare all individual models with one predictor from the five individual difference measures each against the null model with just an intercept. That way, the evidence for the individual null hypotheses could be unambiguously assessed.

**Response 2:** Thank you for pointing out this shortcoming and giving a clear suggestion on how to improve it. We have now run a set of Bayesian linear regressions where we compare the individual models (with only one variable of interest) to the intercept-only model. For Final Choice, we do this for the interaction terms. In addition, in part in response to Reviewer 2’s suggestion to focus more on Final Choice, we also report model-averaged coefficients for Bayesian linear regression models with all variables (including controls) entered for the variables of interest, corroborating previous results.

**Example (p. 26-27):** Further, we conduct exploratory Bayesian analyses (Rouder & Morey 2012; Liang et al. 2008) where we use Bayesian linear regression analyses that draw on Bayesian model averaging (e.g., Hinne et al., 2019, Maier et al., 2022). First, in Table 7, we report results where we compare the individual models with one predictor each (the respective explanatory variable) against the null model with an intercept only. We report Bayes Factor Model Odds for the null for both Main Choice and Final Choice (frequency of donation as well as size of donation). For the analyses in Table 7, we use a uniform model prior.

Table 7—Bayes Factor Model Odds for Null Model

|  | Main Choice | Final Choice (Freq.) | Final Choice (Size) |
| --- | --- | --- | --- |
| Risk Attitude | 7.46 |  |  |
| Ambiguity Aversion | 7.81 |  |  |
| Numeracy | 7.44 |  |  |
| Empathy | 7.89 |  |  |
| Optimism | 3.50 |  |  |
| Warm-Glow Type | 7.94 |  |  |
| Pure Altruistic Type | 5.94 |  |  |
| Condition X Risk Attitude |  | .04 | <.001 |
| Condition X Ambiguity Aversion |  | 13.41 | 7.57 |
| Condition X Numeracy |  | <.001 | <.001 |
| Condition X Empathy |  | <.001 | <.001 |
| Condition X Optimism |  | <.001 | <.001 |
| Condition X Warm Glow |  | 3.82 | 6.23 |
| Condition X Pure Altruism |  | 1.46 | 15.32 |

*Notes:* Bayes Factor Model Odds for the Null Model with Uniform Prior for both the explanatory variables for Main Choice and the interaction terms for Final Choice.

The results in Table 7 indicate that for Main Choice, we have strong additional evidence in favour of a null effect of all variables of interest with Bayes factors of between 3.5 and 7.94. The picture is more complicated with regard to the interaction effects from Final Choice. There, we find that compared to models with ambiguity aversion or altruistic types individually, the null model is much more likely given the data. However, the other explanatory variables do not share this pattern. To further investigate the effect of our main variables on our outcomes of interest, we also report the model averaged coefficients that account for uncertainty over the estimates as well as uncertainty over model choice with all interaction effects and all control variables (age, gender, education, marital status, children, financial well-being, employment, religious affiliation, and religious participation). We report those coefficients as well as their 95% credible intervals that represent a weighted average that is weighted by the posterior probability of predictor inclusion. We use a uniform model prior and a JZS parameter prior with the default r scale of .354.

Table 8—Bayesian Linear Regression Coefficients and 95% Credible Intervals

|  | Final Choices (Freq.) | Final Choice (Size) |
| --- | --- | --- |
| Condition X Risk Attitude | .000 [-.001, .005] | .02 [-.03, .24] |
| Condition X Ambiguity Aversion | .000 [-.001, .005] | -.003 [-.008, .10] |
| Condition X Numeracy | -.01 [-.02, .000] | -.49 [-.89, .000] |
| Condition X Empathy | .000 [-.002, .001] | .001 [-.07, .06] |
| Condition X Optimism | -.001 [-.005, .000] | -.000 [-.03, .08] |
| Condition X Warm Glow | -.004 [-.01, .07] | .19 [-.25, 2.19] |
| Condition X Pure Altruism | .20 [.08, .32] | .90 [.000, 4.66] |

*Notes:* Model averaged coefficients and 95% credible intervals.

The results reported in Table 8 are in line with previous frequentist regression analyses, where we find that pure altruistic donor type predicts donations to probabilistic charities while most other interaction effects do not show any effects or only provide weak evidence in favour of them once all variables are entered into the model. As such, we conclude that the exploratory Bayesian analyses roughly corroborate our previous results.

**Comment 3:** Again, I think the authors did a good job in adding more literature and theoretical arguments to justify why the selected measures should correlate with donation behavior in their study. However, I also noticed that most of the studies they cite are based on different experimental designs. For example, Kleber et al. (2013) found an effect of numeracy on the effect of the number and share of helped people. This feature is not central to the current manipulation of interest. Similarly, in Cettolin et al. (2017) risk preferences are measures with certainty equivalents in decisions from description, whereas the current study uses an experience-based risk elicitation task with a different dependent variable. We already know that behavioral elicitation methods for risk preference correlate little with each other (see Frey et al., 2017). Thus, it is not straight-forward to assume the same underlying relation from different elicitation methods. Ultimately, I think this is not a weakness of the current study, but rather of the field as a whole. I would suggest the authors mention this problem in the discussion and call for more specific cognitive models and theories that build stronger connections between elicitation methods, constructs, and cognitive processes influencing behavior.

References:
Frey, R., Pedroni, A., Mata, R., Rieskamp, J., & Hertwig, R. (2017). Risk preference shares the psychometric structure of major psychological traits. Science Advances, 3(10), e1701381.

**Response 3:** We agree with your comment and have added this reference and made the recommendation you outlined in the discussion section.

**Example (p.30-31):** Additionally, it is important to point that much of the literature that this paper builds on is based on fundamentally different experimental designs. As Frey et al. (2017) have already shown, behavioural elicitation methods for risk preferences often show low correlations between each other, making it not as straight-forward as presented previously to assume a similar underlying relation of different elicitation methods. While we argue that this is not primarily a weakness of the currently, we see it as general issue with the field overall, suggesting that more specific cognitive models and theories that build stronger connections between elicitation methods, constructs, and cognitive processes influencing behaviour would be sorely needed.

**Reviewer #2**

**General Comment:** I appreciate the authors effort to give more weight to Final Choice. The paper has been improved a lot for sure. However, I’m not sure whether the authors understand my major concern well. The point is that you have two moving parts in your Main Choice design. Such a design flaw makes it unable to test your hypotheses and hence is totally invalid.

**General Response:** Thank you very much for giving us the opportunity to revise the paper further and for acknowledging that our previous revisions improved the paper a lot. We have now followed your recommendation in full and are controlling for the amount of donation in all analyses regarding Main Choice and have updated our equivalence test results and Bayesian analyses accordingly.

We would also like to thank you for spelling out your main objection to our Main Choice analysis in much more detail in this round of review. You were right that we had not fully grasped it at the last R&R stage, but we now believe that we have a much better understanding of your main objection to this design. While we hope that you can appreciate that we cannot remove the Main Choice analyses from our paper due to them being our pre-registered hypotheses, we have made four significant changes to our paper that we hope will help address your point. First, we have put even more weight on Final Choice, for example by conducting Bayesian linear regression analyses with, reporting model-averaged coefficients for it (but not for Main Choice). Second, we have followed your recommendation and are now controlling for amount donated in all Main Choice analyses. Third, we now outline your full objection in detail in the discussion section, where we give it substantive space, outline your examples, and detail these challenges for the interpretation of data collected from Main Choice. This is to ensure that all readers are informed about your concerns regarding our design, without compromising on our commitment to conduct, report, and analyse our experiment as pre-registered. Fourth, we have conducted two sets of regressions (one in the appendix, one with same results in this letter) aimed at controlling for your concerns, showing no difference in results. We hope that this goes towards addressing your main concern, though if you have any further specific recommendations, we are happy to follow them too.

**Comment 1:** The reasoning is simple: when one can choose both the type of the charity and the amount to donate, one can choose two different compositions that one is indifferent from. For example, if I’m indifferent between $1/sure thing charity and $0.5/probabilistic charity, I can choose randomly from the two. Overall, you would find roughly 50% choose sure thing and 50% choose probabilistic charity. This means you cannot test the preference for charity type under such a design. This is point one.

**Response 1:** Thank you very much for spelling out your main objection in more detail. We now believe that we better understand your main concern. Your concern is of course legitimate and true. In general, indifference is always a possibility (unless we make some assumptions in terms of the decision maker’s preferences). For example, even if we had given our subjects the choice between donating a fixed amount to the sure thing charity or to the probabilistic charity or donating nothing to either of them, a subject’s choice to donate to one of the two types of charities would not rule out the possibility that the decision maker was indifferent between donating the fixed amount to either of them. Of course, you are right that when the decision maker can choose not just between a sure thing charity and a probabilistic charity indifference but also the amount to be donated, then the indifference situation is more likely to arise than when the amount to be donated is fixed, and the only choice is to which charity to donate or to not donate at all. In the text, the new revised version now explicitly acknowledges the indifference issue you pointed out.

There is, however, a way to delve further into the data to get an idea of the concern you raised. That way involves considering a participant’s choices at Main Choice and Final Choice simultaneously to identify possible cases of indifference. We consider that those who: i) donated to a sure-thing charity in Main Choice but did not donate at all in Final Choice when they were shown a probabilistic charity, might have been indifferent between a strictly positive donation to a sure-thing charity and donating nothing to a probabilistic charity; ii) donated to a probabilistic charity in Main Choice but did not donate at all in Final Choice when they were shown a sure-thing charity, might have been indifferent between a strictly positive donation to a probabilistic charity and donating nothing to a sure-thing charity. Thus, in Appendix F we exclude such subjects from the regressions of Main Choice. The regression estimates replicate findings of the regressions in the main body of the text. See Example 1 below.

Of course, Appendix F’s regressions which are based on the above exclusions do not fully eliminate all the subjects who might have been indifferent between donating a strictly positive amount to one type of charity and a zero donation to the other type of charity. The reasons for this are that: i) subjects who donated to a sure-thing charity at Main Choice and were faced with the decision to donate a sure-thing charity at Final Choice might have been indifferent between donating a positive amount to a sure-thing charity and donating zero to a probabilistic charity at Main Choice, but since they were not faced with a probabilistic charity at Final Choice they did not have the chance to make choices to reveal that indifference; i) likewise, subjects who donated to a probabilistic charity at Main Choice and were faced with the decision to donate a probabilistic charity at Final Choice might have been indifferent between donating a positive amount to a probabilistic charity and donating zero to a sure-thing charity at Main Choice, but since they were not faced with a sure-thing charity at Final Choice they did not have the chance to make choices to reveal that indifference.

This led us to re-run the regressions focusing on different exclusion criteria. We ran the regressions on the subsample of subjects who faced a probabilistic charity at Final Choice, but among those excluded the ones who had donated a positive amount to a sure-thing charity. The excluded subjects made decisions consistent with the indifference concern you stated (i.e., donated a strictly positive amount to a sure-thing charity but made no donation when faced with a probabilistic charity). The non-excluded subjects made decisions that are not consistent with that possibility. The regression estimates broadly replicate findings of the regressions in the main body of the text for this selected sample of subjects. The results for this regression are below (but not in the paper) as the results are the same as in Appendix F.

Table X — Review Response Letter Regression Results for Charitable Giving Behaviour in Main Choice
 Predicting Choice between Sure-Thing and Probabilistic Charities

|  | (X) |
| --- | --- |
| Risk Attitude | .496 (.980) |
|  |  |
| Ambiguity Aversion | .008 (.015) |
|  |  |
| Numeracy | -.002 (.015) |
|  |  |
| Empathy | -.047 (.081) |
|  |  |
| Optimism | .004* (.007) |
|  |  |
| Donor Type |  |
| Warm-Glow | -.014 (.008) |
| Pure Altruism | .002 (.120) |
|  |  |
| Donation (amount) | -.082 (.155) |
|  |  |
| Age | -.001 (.002) |
|  |  |
| Gender | .006 (.005) |
|  |  |
| Education |  |
| Undergraduate degree | .066 (.124) |
| Postgraduate/Professional degree | .215 (.129) |
|  |  |
| Religion |  |
| Protestantism | .056** (.125) |
| Catholicism | -.350 (.152) |
| Islam | -.030 (.163) |
| Judaism | -.115 (.339) |
| Buddhism | -.257 (.538) |
| Hinduism | .307 (.584) |
|  |  |
| Religious Participation | .190 (.203) |
|  |  |
| Marriage Status | .118 (.120) |
|  |  |
| Children | -.067 (.133) |
|  |  |
| Financial Wellbeing | .030 (.067) |
|  |  |
| Employment |  |
| Out of the workforce | -.347 (.229) |
| Part-time employment | .052 (.205) |
| Full-time employment | -.139 (.198) |
|  |  |
| Adj. R^2^ | .009 |
|  |  |
| Sample size | 93 |

*Notes:* OLS regression reporting unstandardised coefficients and standard errors. Outcome variable is charity choice (0 = sure-thing charity, 1 = probabilistic charity). *p<.1, **p<.05, ***p<.01, ****p<.001

We very much hope that these additional analyses and regressions make headway to address the concerns you raised.

Furthermore, as outlined in General Response, we will now present your main objection in significant detail in the discussion section such that readers are fully aware of this design limitation and its impact on the results. We also outline your criticism throughout the paper by pointing out repeatedly that Final Choice ought to be given more weight. See Example 2 and Example 3 below.

**Example 1 (p. 48-49):**

In Appendix Table 11, Model (17), we report the same OLS regression for Main Choice as in Model (1). The main change in this regression is that, in response to the worry that by excluding those that do not donate, we may be biasing our estimates as participants may be indifferent between donating a non-zero amount to one type of charity (say a sure-thing charity) and no donation to a probabilistic charity. As we had additional data on all participants from their choices in Final Choice, we excluded the following participants on top of our standard exclusion criteria. First, we excluded those who donated to a sure-thing charity in Main Choice but did not donate at all in Final Choice when they were shown a probabilistic charity (n=70). Second, we also excluded those who donated to a probabilistic charity in Main Choice but who did not donate at all in Final Choice when they were shown a sure-thing charity (n=5). Overall, we do not find a difference in results which suggests that that our data may go some way towards addressing this worry.

Appendix Table 11—Regression Results for Charitable Giving Behaviour in Main Choice
 Predicting Choice between Sure-Thing and Probabilistic Charities

|  | (17) |
| --- | --- |
| Risk Attitude | .000 (.007) |
|  |  |
| Ambiguity Aversion | -.004 (.006) |
|  |  |
| Numeracy | -.005 (.030) |
|  |  |
| Empathy | -.001 (.003) |
|  |  |
| Optimism | -.004 (.004) |
|  |  |
| Donor Type |  |
| Warm-Glow | -.012 (.065) |
| Pure Altruism | -.086 (.076) |
|  |  |
| Donation (amount) | -.001 (.001) |
|  |  |
| Age | .004 (.002) |
|  |  |
| Gender | -.002 (.062) |
|  |  |
| Education |  |
| Undergraduate degree | .068 (.064) |
| Postgraduate/Professional degree | -.017 (.069) |
|  |  |
| Religion |  |
| Protestantism | -.116 (.084) |
| Catholicism | -.045 (.089) |
| Islam | -.111 (.171) |
| Judaism | -.198 (.412) |
| Buddhism | -.151 (.318) |
| Hinduism | .319 (.303) |
|  |  |
| Religious Participation | .057 (.109) |
|  |  |
| Marriage Status | .044 (.062) |
|  |  |
| Children | .000 (.069) |
|  |  |
| Financial Wellbeing | .007 (.030) |
|  |  |
| Employment |  |
| Out of the workforce | -.213 (.122) |
| Part-time employment | -.005 (.104) |
| Full-time employment | -.099 (.096) |
|  |  |
| R^2^ | .081 |
|  |  |
| Sample size | 232 |

*Notes:* OLS regression reporting unstandardised coefficients and standard errors. Outcome variable is charity choice (0 = sure-thing charity, 1 = probabilistic charity). *p<.1, **p<.05, ***p<.01, ****p<.001

**Example 2 (p. 31-32):** Moreover, there is an additional reason to significantly favour the results of Final Choice over those of Main Choice throughout the interpretation of this paper’s data. This is because the design of Main Choice has some inherent flaws that make interpretation of results difficult. This is because when participants make choices between two charities, they can make choices between compositions that they are indifferent between. For example, any one donor may be indifferent between a donation of £0.25 to a sure-thing charity and a donation of £0.10 to a probabilistic charity. In this scenario, the donor would choose randomly between the two. This in itself makes it difficult to cleanly identify a preference for one charity type or the other in the design that we have pre-registered and analysed in this paper. Based on this reason, we argue that the results from Final Choice should be given greater weight compared to Main Choice throughout this paper.

This concern is even more pointed if one considers that it may be the case that donors could be indifferent between donating a positive amount to a sure-thing charity and no donation to a probabilistic charity. As before, if they are indifferent between the two and choose randomly, this inhibits straightforward interpretation of our results. This is because we exclude all participants who choose not to donate (to understand their behaviour between these two options). Further, because it is quite plausible that, given the distribution of donation choices (most people who donate donate to a sure-thing charity), we may be excluding significantly more people favouring probabilistic charities but not sure-thing charities from those who are indifferent between a non-zero donation to a sure-thing charity and a zero donation to a probabilistic (and thus choose randomly between the two).

Some of these concerns have been addressed ex-post in this paper, for example by controlling for amount donated in the analyses of Main Choice that we did not initially pre-register. While this goes some way towards addressing this concern, we argue that some fundamental design constraints of the set-up that we chose remain. As such, we have put a higher emphasis on our analyses of Final Choice (which do not fall prey to the same structural challenges) throughout this paper and argue that one ought to be generally cautious in interpreting the results of Main Choice. However, given that it was pre-registered, we continue to report it fully (and where we deviate from the pre-registered protocol, we document this in detail and provide the original analyses in the appendix). We hope that this discussion, highlighting these issues in detail, properly contexualises the results for readers.

**Example 3 (p. 15):** This condition [*Final Choice*] controls for a number of potential confounds in Main Choice which allows for it to answer the paper’s central question more directly and cleanly, though it being the last task of the experiment, we cannot rule out potential order effects.

**Comment 2:** Point two, suppose people are more likely to donate more under sure-thing charity, then when you excluded 0 donation as you preregistered, you exclude more people who chose probabilistic charity (you can test this with your data). Th reason is that there must be some people who are indifferent between $positive amount/sure thing charity and $0/probabilistic charity, and these people chose randomly between the two. In the observed results, you excluded those who chose probabilistic charity but not the other type.

**Response 2:** Thank you again for further clarifications on this. As outlined above, we now discuss in detail your objection in the discussion section, including the examples you have provided here to ensure we do not misrepresent your main concern, while also offering regressions that hopefully go some way towards addressing this concern. Further, in order to make sure we are not missing something in our exchanges, we re-read our paper line by line. In doing so, we have realised that we have not made clear enough in the main paper that participant choices in Main Choice are between donating a non-zero amount to either of the two charities or not donating at all. Participants cannot choose a charity and then enter ‘0’. We have now made this clear in the paper at multiple points and apologise for this oversight in our previous revision.

**Example (p. 5, 9, 14, 29):** In attempting the construction of an externally valid donation choice that allows us to capture the difference between sure-thing charities and probabilistic charities in actual charities that participants can donate money to, our main outcome variables of interest are (i) the choice between two real charities, one sure-thing charity and one probabilistic charity and (ii) the choice about one of those charities that has been randomly selected. Both of these charitable decision-making scenarios have strengths and weaknesses from an experimental design perspective, but we hope that they jointly allow us to better understand the role of individual differences in charitable decision-making scenarios like these. We outline the main weakness of (i) in the discussion section and argue that, overall, (ii) is a cleaner design.

Lastly, one may also be interested in donation behaviour not between these two types of charities, but rather just in the context where potential donors are presented with one such charity. This may reduce the chance of additional confounds (like worrying that the design that presents two charities is artificial in its dichotomous presentation; after all, most naturalistic decisions are not decisions between two distinct choices). It also is overall a cleaner design that brings with it less drawbacks regarding interpretation of results.

In both cases, we collect data on whether they donate at all, if they do, which charity they choose in Main Choice (participants can choose up to one charity), and how much they donate. In other words, participants can donate a non-zero amount to either charity or not donate at all (i.e. one cannot select a charity and choose to donate ‘0’).

We have some evidence in favour of this worry as at least some of the pre-registered factors predict directionally as expected in Final Choice, where only one charity was presented to participants. As such, it may be justified to put more interpretative emphasis on the results from Final Choice compared to Main Choice.

**Comment 3:** Point 3, because of the above issue, if you regress charity type preference on other variables, you have to control for the amount donated, as an ex-post control, because you did not control for the donate amount in the experiment. I’m not clear what endogeneity this may cause. But if there is any, I don’t believe the data from Main Choice can prove anything. There is no problem if you want to report the analyses as you preregistered. But a preregistration doesn’t mean your design and analysis (Main Choice) is not problematic.

**Response 3:** Thank you very much for clarifying your comment from last time. As a result of your explanation of your main objection above, we have now followed your recommendation in full by controlling for the amount donated as an ex-post control for all analyses of Main Choice.

**Example (p. 20-22):** First, we investigate general donation behaviour relating to Main Choice. The results presented in Table 3 speak to the central null hypotheses #1, #2, and #3. Model (1) reports the results for the Main Condition. The outcome variable is the type of charity conditional on a donation being made, with 0 being coded as the sure-thing charity and 1 as the probabilistic charity. The gender variable is coded 1 for female, all other categorical variables are coded 1 for the affirmative. As outlined above, the risk attitude measure is a discrete variable of the number of boxes opened, the ambiguity aversion is the result of the subtraction of the reservation prices. All other scales are the sum of the (re-reversed) individual items. As specified in our pre-registration, we report main regression results for binary outcomes using an OLS model and also for the corresponding logit model as a robustness check in Appendix B (Appendix Table 2) to check for sensitivity to functional form choice, where we find no impact of model choice. We also report a robustness check in Appendix B (Appendix Table 3) where we report a regression with random effects at the stimulus level, finding that our null result is also robust to this model choice.

Table 3—Regression Results for Charitable Giving Behaviour in Main Choice
 Predicting Choice between Sure-Thing and Probabilistic Charities

|  | (1) |
| --- | --- |
| Risk Attitude | .000 (.005) |
|  |  |
| Ambiguity Aversion | -.003 (.005) |
|  |  |
| Numeracy | .004 (.024) |
|  |  |
| Empathy | <.001 (.003) |
|  |  |
| Optimism | -.004 (.003) |
|  |  |
| Donor Type |  |
| Warm-Glow | -.014 (.051) |
| Pure Altruism | -.062 (.064) |
|  |  |
| Donation (amount) | -.001 (.001) |
|  |  |
| Age | .003 (.002) |
|  |  |
| Gender | .013 (.050) |
|  |  |
| Education |  |
| Undergraduate degree | .044 (.051) |
| Postgraduate/Professional degree | -.020 (.055) |
|  |  |
| Religion |  |
| Protestantism | -.082 (.067) |
| Catholicism | .012 (.073) |
| Islam | -.101 (.122) |
| Judaism | -.163 (.379) |
| Buddhism | -.105 (.287) |
| Hinduism | .378 (.276) |
|  |  |
| Religious Participation | .041 (.089) |
|  |  |
| Marriage Status | .045 (.050) |
|  |  |
| Children | -.007 (.054) |
|  |  |
| Financial Wellbeing | .005 (.024) |
|  |  |
| Employment |  |
| Out of the workforce | -.154 (.097) |
| Part-time employment | .003 (.085) |
| Full-time employment | -.054 (.079) |
|  |  |
| R^2^ | .064 |
|  |  |
| Sample size | 307 |

*Notes:* OLS regression reporting unstandardised coefficients and standard errors. Outcome variable is charity choice (0 = sure-thing charity, 1 = probabilistic charity). *p<.1, **p<.05, ***p<.01, ****p<.001

We find that none of the main variables nor the demographic control variables predict charity choice in the Main Condition. We can straightforwardly conclude from Model (1) that we do not have evidence to reject the null hypotheses #1 and #2 as none of the independent variables meaningfully predict donor behaviour. However, note that Model (1) includes the control variable of amount donated that was not pre-registered but suggested by a helpful anonymous reviewer. For the pre-registered regression model without this control with no difference in results, please see Appendix A.
