# Supplementary material for: Sure-thing vs. probabilistic charitable giving: Experimental evidence on the role of individual differences in risky and ambiguous charitable decision-making
Source: PLoS One. 2022 Sep 22;17(9):e0273971. doi: 10.1371/journal.pone.0273971 (PMC9499298; doi:10.1371/journal.pone.0273971)
Supplement: Attachment — Submitted filename: Response to Editor.docx [file pone.0273971.s014.docx]

Dear Editor,

Below we outline our response to your last comment.

**Comment:** Please explain the limitations about this kind of study using web-based sample.

**Response:** We agree that we have not properly stated this limitation before and now do so explicitly in the revised manuscript.

**Example (p. 33):** A second potential limitation is the use of a web-based sample from Prolific. While we have tried to counterbalance this concern by relying on a representative sample (at least along the dimensions of age, sex, and ethnicity), there may still be some dimension along which our sample is not representative of the population as a whole. This, in turn, means that that there may be some external validity worries inherent in using this sample, which may impact generalisability of our results.
